# Prevalence of antimicrobial resistance and its clinical implications in Ethiopia: a systematic review

**DOI:** 10.1186/s13756-021-00965-0

**Published:** 2021-12-03

**Authors:** Derbew Fikadu Berhe, Getachew Tesfaye Beyene, Berhanu Seyoum, Meseret Gebre, Kassa Haile, Mulugeta Tsegaye, Minyahil Tadesse Boltena, Emawayish Tesema, Taddele Cherinet Kibret, Mulatu Biru, Dawd S. Siraj, Daniel Shirley, Rawleigh Howe, Alemseged Abdissa

**Affiliations:** 1 College of Health Sciences, School of Pharmacy, Mekelle University, Mekelle, Ethiopia; 2 Armauer Hansen Research Institute, P.O. Box: 1005, Addis Ababa, Ethiopia; 3 Department of Internal Medicine, ALERT Hospital, Addis Ababa, Ethiopia; 4 Department of Statistics, Addis Ababa University, Addis Ababa, Ethiopia; 5 Division of Infectious Diseases, Department of Medicine, School of Medicine and Public Health, University of Wisconsin, University of Wisconsin- Madison, Madison, USA

**Keywords:** Antibacterial resistance, Antibiotic resistance, Drug resistance, Systematic review, Ethiopia

## Abstract

**Background:**

Antimicrobial resistance is one of the major public health challenges in Ethiopia. However, there is no comprehensive summary of existing AMR data in the country.

**Aim:**

To determine the prevalence of antimicrobial resistance and its clinical implications in Ethiopia.

**Methods:**

A systematic literature search was performed on the PubMed/Medline database. Original studies on antimicrobial resistance conducted in Ethiopia between 1st January 2009 and 31st July 2019 were included. The outcome measure was the number of isolates resistant to antimicrobial agents in terms of specific pathogens, and disease condition. Data was calculated as total number of resistant isolates relative to the total number of isolates per specific pathogen and medication.

**Results:**

A total of 48,021 study participants enrolled from 131 original studies were included resulting in 15,845 isolates tested for antimicrobial resistance. The most common clinical sample sources were urine (28%), ear, nose, and throat discharge collectively (27%), and blood (21%). All the studies were cross-sectional and 83% were conducted in hospital settings. Among Gram-positive bacteria, the reported level of resistance to vancomycin ranged from 8% (*Enterococcus* species) to 20% (*S. aureus*). *E. coli, K. pneumoniae* and *P. aeruginosa* were the most common Gram-negative pathogens resistant to key antimicrobial agents described in the national standard treatment guideline and were associated with diverse clinical conditions: urinary tract infections, diarrhea, surgical site infections, pneumonia, ocular infections, and middle ear infections.

**Conclusion:**

Overall, there is a high prevalence of antimicrobial resistance in Ethiopia. Empirical treatment of bacterial infections needs to be guided by up-to-date national guidelines considering local antimicrobial susceptibility patterns. Equipping diagnostic laboratories with culture and drug susceptibility testing facilities, and establishing a strong antimicrobial stewardship program should be high priorities.

**Supplementary Information:**

The online version contains supplementary material available at 10.1186/s13756-021-00965-0.

## Background

Inappropriate use of antimicrobial agents contributes to the development and spread of antimicrobial resistance (AMR) and hinders the global effort to mitigate infectious diseases [1, 2]. The ineffectiveness of antibiotics in killing microbes, non-adherence to standard prescription, complex human mobility, poor healthcare seeking behavior, and shift in demography and other factors contribute to microorganisms’ adaptation to antibacterial agents [3].

According to a WHO report, there is scarcity of usable data to guide policy recommendations on AMR, especially in the Africa region. This is due to sizable problems associated with data inadequacy as only few countries collect and report continuous surveillance of drug resistance [4, 5]. Laboratories are often poorly equipped to test for and document AMR to meet the goals outline in the WHO global strategy for the control of AMR through laboratory-based surveillance as an essential tool document [6–9]. The few available reports revealed that the WHO African region accounts for increased incidence of the AMR worldwide with significant reports of resistance observed for *Vibrio cholerae*, *Shigella dysentery*, *Salmonella typhi*, *Neisseria gonorrhoea*, *Mycobacterium tuberculosis*, *Plasmodium falciparum* and HIV-type I [10, 11].

In sub-Saharan Africa (SSA), the situation of AMR has become more complicated due to poor hygiene, inadequate clean water supply, conflicts, and increasing number of immune-compromised people through time. Most studies conducted on AMR in East Africa have been hospital-based and cross-sectional in design and limited to bloodstream infections, illustrating an incomplete understanding of the range of clinical scenarios impacted by AMR. Moreover, among many of the easily accessible and affordable drugs, such as penicillin G, co-trimoxazole, ampicillin, and amoxicillin resistance approached 100% [12, 13], underscoring the severity of the problem.

Despite efforts to tackle the AMR problem in Ethiopia, barriers remain, including lack of sufficient antimicrobial stewardship programs at the health facilities, lack of updated national and/or facility-based treatment guidelines informed by local antimicrobial susceptibility pattern, insufficient laboratory facilities and resources, and poor pharmacovigilance systems [14]. Fragmented studies on AMR have been conducted in different regions of Ethiopia. However, these data have not been systematically synthesized to generate actionable evidence that could influence policy modifications. Therefore, this study is aimed at systematically reviewing the prevalence of antimicrobial resistance, the empiric uses of antibiotics and critically examining the utility of the national and international treatment guidelines in Ethiopian context.

## Methods

### Search strategy

Following the Preferred Reporting Items of Systematic Reviews and Meta-Analyses (PRISMA) guidelines[15], a systematic literature search was performed using PubMed/Medline. Search terms were grouped into two queries (Ethiopia and antibacterial resistance related terms), using the Boolean operators, ‘or’ [within a query] /’and’ [between the two main queries] 'or' [between antibacterial resistance terms]. Antibacterial resistance search terms include “antibiotic resistance”, “antibiotic susceptibility”, “antibiotic sensitivity”, “antimicrobial susceptibility”, “antimicrobial sensitivity”, “antimicrobial resistance”, “antibacterial resistance”, “resistance”, “bacterial”, “Drug Resistance”, “Drug Resistance, Bacterial”. Studies published in a 10-year time span from 1st January 2009 and 31st July 2019 were included.

### Eligibility criteria

We included original studies written in English with full text access that evaluated antimicrobial resistance in Ethiopia. Reviews, letters to editors, conference abstracts, commentaries, and articles on *Mycobacterium tuberculosis* drug resistance were excluded. In addition, studies that did not have quantifiable AMR data, AMR studies on animals, plants, environmental studies that did not involve human and/or healthcare facilities, and systematic reviews and meta-analysis were also excluded.

### Study selection and data extraction

Search results from PubMed/Medline were exported to Microsoft Access and prepared for a cascade of screening*.* Titles/abstracts were independently screened by two investigators (TC and MTB), and double checked by DFB. Full text screening was performed by two groups of investigators (a group led by DFB; another group led by GTB). Once full text screening was completed, an electronic case report form/data abstraction tool was prepared on Microsoft Access. The data extraction process involved three steps to minimize potential errors. First, the two groups of investigators led by DFB and GTB extracted relevant information from each included paper. Secondly, the two groups of investigators double checked each other’s work, finally DFB, GTB, MTB and TC double checked the final data. There were five rounds of scientific meetings among all investigators for consensus for any differences in article screening, selection and data abstraction.

Data was extracted using a data collection form that included information on sample source, sample size, geographic region, biological age group, clinical sample type, the study setting/type of healthcare facility, disease/clinical condition, antibacterial agent tested for AMR, bacterial species tested for AMR, total number of isolates per paper, AMR data in absolute numbers for each antibacterial agent and bacterial species (Additional file 1: Table S1).

Studies with specific age groups were presented as pediatric/neonate, adult or not specific. Clinical sample type was categorized as blood; stool/anorectal swab; urine; ear, nose and throat discharge/swab; wound (swab/discharge); urethral swab/discharge; vaginal swab/discharge; cerebrospinal fluid; and others (air or inanimate objects at the healthcare setting). Study settings were classified as hospital, health centers, and community or laboratory databases. For disease/clinical condition, major categories were surgical site infection, urinary tract infection, diarrhea, sepsis, pneumonia, wound infection, sexual transmitted infection, ocular infection, ear infection, and gastroenteritis. Studies that did not clearly specify disease condition were labeled as ‘’no disease or not specific’’.

### Reporting of the review

The PRISMA guideline has been used in this report to ensure the clarity, transparency and quality of the AMR evidence synthesis. Both the PRISMA diagram and the PRISMA check lists have been utilized in the study, where appropriate.

### Assessment of the quality of the included studies

To assess the quality of included papers, we used the Newcastle–Ottawa Scale (NOS) adapted for cross-sectional studies [16]. The scale containing items on representativeness of the study population included in the studies (sampling method, sample size, response rate and demographics), the methods used to test the AMR and the way AMR data was described. The sum score of all seven items was set at 10 with a maximum score of two for item 4, 5, and 6; and 1 point for all others.

### Outcome measure

Data on antimicrobial resistance was extracted from each study. Definitions of the term resistance, intermediate and susceptible were directly taken from each study, based on the author’s interpretation. We computed the ‘AMR’ by taking absolute numbers reported by each study. AMR data was extracted per the antimicrobial agent and per the specific pathogen studied. Total resistant numbers of isolates were extracted from each paper. Numbers of resistant isolates were calculated for studies providing AMR data as percent of the total isolates or sensitivity of antimicrobial agents. AMR data is presented for most commonly reported pathogens (Gram-positive and Gram-negative bacteria (Tables 2, 3).

### Statistical analysis

This study focused on evidence synthesis on the prevalence of resistance patterns of bacterial pathogens in Ethiopia with emphasis on clinical implication. The AMR data among studies were very heterogeneous in terms of antimicrobial agents and pathogens. Therefore, descriptive statistics were used to describe the prevalence of AMR with special emphasis on bacterial species, antimicrobial agents, and disease conditions. Implication of the current findings is described in reference to Ethiopian Standard Treatment Guideline (STG) and inferred from international standards (UpToDate) [17].

### Data presentation

Data was stratified according to clinical condition, microorganism and antimicrobial agent. Recommended antibiotics according to different guidelines are indicated, and for each recommended antibiotic the percentage of resistance among the total isolates were presented for the given clinical condition.

## Results

### Study characteristics

**Fig. 1:**
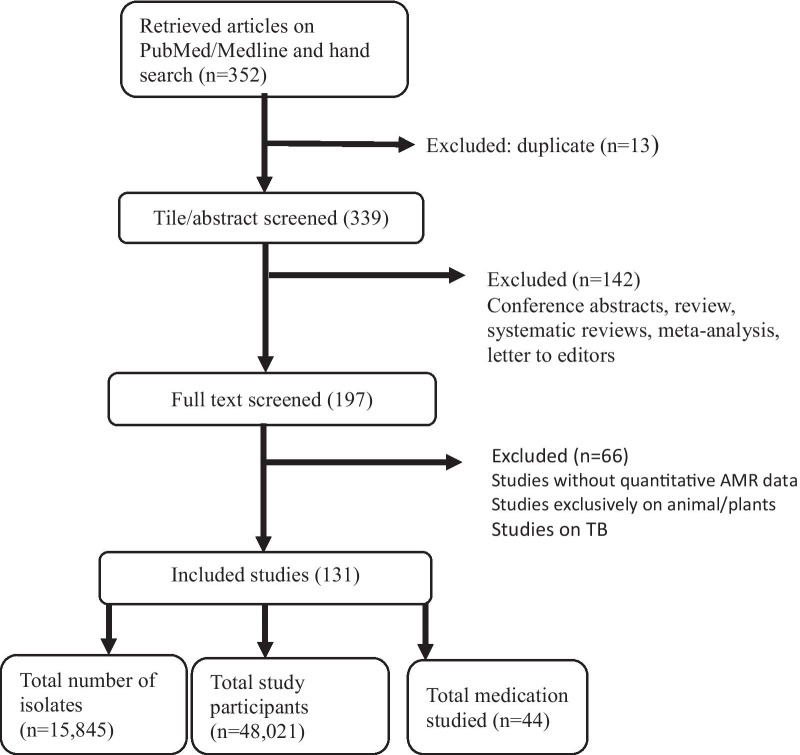
A flow diagram showing study selection and data extraction

Out of 345 reviewed publications, 131 papers met our inclusion criteria (Fig. 1). Overall, the review included 48,021 study participants and 15,845 total bacterial isolates. Seventy-six papers (57%) were age specific, of which 26 (34%) were conducted on all age groups (Tables 1, 2). The total number of pediatrics/neonates included in the review were 8780 (34%) and adults 16,792 (66%). Ninety four percent of the reviewed studies involved human subjects and the majority (83%) of the studies were conducted in a hospital setting, with urine samples constituting the highest (28%) among sample sources. One third of the reviewed papers (n = 48) did not have disease specific studies. From studies with specific diseases, the most common clinical conditions were urinary tract infection (n = 26), diarrhea (n = 17) and wound infection (n = 10) (Table 1 and Additional file 1: Table S1).

**Table 1:** General summary of all the papers reviewed in terms of their source of sample, age group, clinical sample type, study region, study setting, and clinical condition

Description	Papers, n (%)	Number of isolates (n)
*Source of sample*		
Human	126 (95)	15,092
Environmental	5 (4)	425
Inanimate objects	4 (3)	307
*Age group*		
Adult	58 (44)	7886
Pediatrics/neonate	44 (33)	7065
No age group provided	57 (43)	6101
*Clinical sample type*		
Urine	39 (29)	4783
Ear, nose and throat discharge/swab	37 (27)	7396
Stool /anorectal swab	29 (21)	2774
Blood	28 (21)	228
Wound (swab/discharge)	24 (18)	3732
Vaginal swab/discharge	9 (7)	1200
CSF	6 (4)	679
Urethral swab/discharge	5 (4)	1357
Others	22 (16)	2617
*Study setting*		
Hospital	110 (83)	9572
Health center	16 (12)	1327
Laboratory database	14 (101)	4877
Community	11 (8)	1537
*Disease/clinical condition*		
UTI	26 (20)	3302
Diarrhea	18 (14)	1174
Wound infection	10 (8)	1593
Ear infection	9 (7)	3100
Eye infection	7 (5)	746
Fever of undefined disease	7 (5)	464
SSI	6 (5)	558
Sepsis	5 (4)	333
Pneumonia	3 (2)	308
STI and genital area infections	5 (4)	257
Other diseases	13 (10)	1671
No disease or not specific	33 (25)	3398

CSF, cerebrospinal fluid; SNNPR, Southern Nations; Nationalities, and People's Region; UTI, urinary tract infection; STI, sexually transmitted infection; SSI, surgical site infection

**Table 2:** Antimicrobial resistance profile of frequently isolated Gram-positive bacteria, Ethiopia

Antibacterial agent	*S. aureus* *n* (%)	*S. pneumonia* *n* (%)	*S. pyogenes* *n* (%)	*S. agalactae* *n* (%)	*CoNS* *n* (%)	*Enterococcus spp.* *n* (%)
Trimethoprim-sulfamethoxazole	1272 (44)	300 (40)	54 (57)	1 (11)	439 (51)	24 (26)
Amoxicillin	706 (69)	11 (23)	10 (24)	1 (50)	233 (57)	16 (33)
Amoxicillin-clavulanic acid	259 (26)	20 (65)	21 (33)		101 (23)	19 (54)
Ampicillin	1267 (70)	112 (38)	40 (48)	20 (9)	358 (57)	103 (55)
Azithromycin	87 (47)	7 (28)		26 (15)	88 (48)	20 (80)
Cefixime	11 (26)		1 (50)		3 (23)	
Cefotaxime	120 (41)	2 (100)		14 (34)	30 (34)	10 (40)
Cefoxitin	349 (27)	1 (8)	2 (12)		113 (29)	
Ceftazidime	145 (41)	1 (13)	1 (10)		62 (37)	2 (100)
Ceftriaxone	514 (32)	40 (10)	26 (26)	59 (28)	238 (31)	12 (19)
Cefuroxime	74 (26)	1 (11)			11 (48)	6 (46)
Cephalexin	19 (66)	3 (38)			8 (67)	2 (12)
Cephalothin	299 (41)				16 (15)	
Chloramphenicol	968 (40)	132 (18)	21 (29)	28 (12)	277 (42)	53 (30)
Ciprofloxacin	479 (20)	32 (10)	12 (14)	24 (13)	192 (21)	55 (33)
Clarithromycin	50 (70)				66 (79)	
Clindamycin	403 (25)	6 (21)	16 (25)	61 (24)	88 (18)	9 (27)
Cloxacillin	397 (78)		1 (33)		44 (66)	
Doxycycline	268 (38)	26 (26)	15 (37)		122 (31)	30 (64)
Erythromycin	1292 (45)	155 (23)	23 (22)	51 (20)	286 (39)	52 (37)
Gentamicin	813 (29)	58 (27)	19 (25)	1 (50)	210 (26)	49 (26)
Methicillin	78 (35)		1 (14)		30 (28)	1 (50)
Nalidixic acid	31 (45)			1 (50)	25 (33)	2 (22)
Nitrofurantoin	72 (22)		2 (17)		43 (20)	8 (20)
Norfloxacin	232 (26)	16 (33)	9 (23)	1 (50)	77 (19)	17 (28)
Oxacillin	361 (47)	88 (28)	1 (7)		78 (35)	13 (69)
Penicillin	1960 (82)	101 (25)	24 (24)	43 (17)	331 (55)	46 (43)
Tetracycline	1348 (53)	308 (41)	32 (43)	168 (71)	322 (47)	101 (61)
Tobramycin	26 (29)				3 (16)	7 (30)
Vancomycin	208 (20)			35 (14)	26 (10)	10 (8)
Range (%)	20–89	0–100	0–57	0–71	10–79	0–100
Average resistance (%)	42	24	22	23	37	37

n = count of resistant bacterial isolates. % = percent of resistant bacterial isolates

### Study quality

All studies were descriptive cross-sectional surveys. The mean quality score of the papers was 7.5 (SD 1.4). The included papers scored poorly with regards to sample size calculation (item 1), sampling methods (item 2) and tool validation (item 7 of our quality score tool).

### Antibiotic resistance profile of Gram-positive bacteria

The highest number of Gram-positive isolates were *S. aureus* (n = 3470)*, S. pneumoniae* (n = 775)*, and S. pyogenes* (n = 118)*.* For *S. aureus*, resistance to anti-Staphylococcal penicillins (oxacillin, cloxacillin, methicillin) ranged from 35 to 47% and high minimal inhibitory concentration to vancomycin was reported in 20%. Resistance to doxycycline (38%), tetracycline (52%), and TMP/SMX (44%) were also alarmingly high. For *S. pneumoniae*, resistance to penicillin was noted at 25%, ceftriaxone at 10% and azithromycin at 28% (few isolates tested). For *S. pyogenes*, resistance to penicillin was noted at 24% and ceftriaxone at 26%. *S. pyogenes* resistance to amoxicillin/clavulanic acid (32%), TMP/SMX (56%) and tetracycline (42%) were documented. Vancomycin resistance in Gram-positive bacteria varied from 8 to 20%. Over 50% of enterococcus species were resistant to ampicillin but vancomycin resistant enterococci prevalence was 8% (Table 2).

### Antibiotic resistance profile of Gram-negative bacteria

Among Gram-negative bacteria, *E. coli* (n = 3151)*, P. aeruginosa* (n = 857) *and K. pneumoniae* (n = 480) were the most common isolates with high resistance to the commonly used antimicrobial agents. Over 50% of *K. pneumoniae, E. coli, Proteus, Enterobacter species*, *Citrobacter*, *Acinetobacter and Pseudomonas* isolates were resistant to amoxicillin/clavulanic acid. Ceftazidime resistance was over 50% except for *Pseudomonas, Proteus* and *Citrobacter.* Resistance to ceftriaxone ranged between 38–74%*.* In addition, 14% of *K. pneumoniae,* 39% of *P. aeruginosa* and 35% of the *Acinetobacter* species isolates were carbapenemase resistant (Table 3). For *E. coli*, the resistance rate for trimethoprim-sulfamethoxazole was 59%, ceftriaxone 38%, ciprofloxacin 26%, piperacillin-tazobactam 44%, and cefepime 25%. For *P. aeruginosa*, the resistance rate for ciprofloxacin was 20%, cefepime 56%, gentamicin 27%, piperacillin-tazobactam 33% (small numbers), and meropenem 39% (small numbers). For *K. pneumoniae*, the resistance rate for trimethoprim-sulfamethoxazole was 66%, ceftriaxone 56%, piperacillin-tazobactam 52%, cefepime 56%, and meropenem 14% (small numbers).

**Table 3:** Antimicrobial resistance profile of frequently isolated Gram-negative bacteria, Ethiopia

Antibacterial agent	*K. pneumoniae n *(%)	*Klebsiella spp. n *(%)	*E. coli n *(%)	*Enterobacter spp. n *(%)	*Proteus spp. n *(%)	*Salmonella spp. n *(%)	*Shigella spp. n *(%)	*Citrobacter spp. n *(%)	*P. aeruginosa n *(%)	*Acinetobacter spp. n *(%)	*Campylobacter spp. n *(%)
Trimethoprim-sulfamethoxazole	242 (66)	275 (62)	1531 (59)	99 (48)	539 (55)	63 (30)	71 (49)	144 (65)	367 (67)	87 (67)	66 (49)
Amikacin	17 (13)	13 (10)	36 (10)	4 (7)	9 (8)			19 (49)	20 (11)	47 (66)	
Amoxicillin	76 (83)		526 (71)	60 (64)	240 (68)	54 (63)	93 (85)	72 (77)	162 (88)	12 (86)	32 (80)
Amoxicillin-clavulanic acid	167 (61)	128 (57)	794 (52)	29 (64)	138 (64)	20 (11)	13 (20)	57 (70)	178 (78)	30 (44)	16 (36)
Ampicillin	305 (93)	245 (75)	1452 (83)	63 (66)	570 (77)	115 (43)	102 (84)	107 (74)	329 (86)	47 (60)	74 (53)
Azithromycin			11 (32)		11 (44)		10 (34)				
Aztreonam	29 (97)	44 (96)	116 (97)	9 (100)	4 (100)				16 (59)		
Cefepime	59 (53)	7 (23)	109 (35)	15 (48)	19 (38)			10 (37)	37 (56)	41 (85)	
Cefotaxime	57 (56)	102 (54)	270 (45)	21 (42)	148 (50)	8 (36)		21 (42)	89 (48)	56 (68)	
Cefoxitin	34 (43)	5 (8)	120 (37)	6 (50)	8 (50)	13 (8)	6 (27)	11 (55)	15 (42)		
Cefpirome	24 (100)	2 (50)	20 (100)	3 (100)							
Cefuroxime	9 (50)		59 (44)					2 (50)	1 (33)		
Ceftazidime	143 (57)	71 (63)	384 (52)	61 (70)	48 (35)			17 (19)	69 (34)	66 (65)	
Ceftriaxone	201 (56)	154 (40)	677 (38)	80 (40)	1076 (72)	38 (13)	18 (23)	80 (32)	275 (50)	87 (73)	10 (10)
Cefuroxime	14 (50)	30 (73)	84 (41)	2 (33)	29 (55)	2 (100)		10 (50)	12 (55)	31 (86)	
Cephalexin			50 (53)	6 (100)	7 (78)			3 (33)	8 (89)		
Cephalothin	13 (76)	13 (36)	237 (49)	26 (65)	246 (69)	28 (18)	29 (63)	11 (55)	70 (59)	2 (7)	91 (89)
Cephazolin	9 (90)		22 (92)		2 (40)				6 (100)		
Chloramphenicol	136 (53)	153 (45)	767 (36)	64 (35)	334 (48)	62 (20)	80 (45)	111 (61)	289 (53)	31 (39)	24 (18)
Ciprofloxacin	159 (37)	112 (25)	629 (26)	27 (13)	73 (11)	31 (9)	17 (12)	56 (25)	149 (20)	82 (61)	20 (13)
Clindamycin	4 (17)	4 (31)	18 (23)	5 (56)	9 (23)			4 (57)	11 (28)	5 (16)	28 (34)
Doxycycline	24 (75)	67 (67)	222 (58)	8 (35)	177 (68)	19 (59)	5 (83)	30 (70)	186 (73)	36 (67)	16 (20)
Erythromycin	21 (27)	77 (76)	384 (71)	57 (80)	230 (46)	26 (58)	23 (88)	27 (73)	222 (73)	25 (44)	45 (30)
Gentamicin	185 (47)	219 (42)	852 (29)	57 (26)	375 (34)	33 (11)	34 (28)	79 (33)	217 (27)	82 (61)	41 (30)
Imipenem		1 (2)	7 (5)						15 (37)		
Meropenem	9 (14)	2 (4)	7 (4)	1 (7)					14 (39)	16 (35)	
Moxifloxacin	24 (80)		11 (85)	7 (78)	1 (25)				3 (100)		
Nitrofurantoin	30 (48)	81 (34)	151 (14)	12 (35)	74 (36)	45 (27)		28 (57)	50 (41)	4 (29)	
Norfloxacin	41 (42)	60 (32)	182 (21)	7 (16)	84 (17)	24 (18)	11 (8)	22 (26)	55 (24)	4 (9)	10 (11)
Oxacillin		40 (98)	70 (85)	4 (50)	5 (83)	4 (100)		5 (31)	2 (100)	2 (100)	
Penicillin		71 (83)	160 (78)	3 (33	51 (46)			2 (15)	34 (49)	10 (29)	
Piperacillin-tazobactam	25 (52)	20 (43)	112 (44)						2 (33)		
Tetracycline	203 (59)	173 (56)	1668 (74)	97 (51)	682 (85)	91 (34)	93 (70)	129 (68)	336 (81)	28 (56)	54 (36)
Tobramycin	47 (60)	46 (69)	205 (63)	10 (59)	25 (48)			10 (37)	15 (32)		
Range (%)	13–100	2–98	4–100	7–100	8–100	8–100	8–88	15–77	11–100	7–100	10–89
Average resistance (%)	57	48	50	51	51	37	48	49	55	54	36

n = count of resistant bacterial isolates. % = percent of resistant bacterial isolates

*E. coli, K. pneumoniae, P. aeruginosa, and S. aureus* were the most frequently isolated causative agents of UTI (Table 4). Referring to the Ethiopian standard treatment guideline (STG) for UTI, almost all recommended first and second line antimicrobial agents for UTI showed a relatively high degree of resistance for the most common causative pathogens: ampicillin (85%), gentamicin (32%), amoxicillin (80%), cephalexin (62%) and trimethoprim-sulfamethoxazole (57%) (Tables 4 and 5).

**Table 4:** Most common clinical conditions with the main pathogens and their antimicrobial resistance profile, Ethiopia

Clinical condition	Most frequently isolated pathogens	Antimicrobial profile: total isolates (% resistance)										
		TSX	AMP	AMC	AMC-ClA	CIP	CRO	GCN	CLO	NOR	OX/MET	CARBS
Urinary tract infection	*E. coli*	1009 (60)	898 (87)	222 (83)	484 (46)	845 (28)	84 (35)	1210 (28)	650 (41)	268 (22)	–	–
	*K. pneumoniae*	92 (52)	131 (92)	24 (88)	83 (48)	120 (31)	86 (24)	111 (38)	65 (54)	38 (42)	–	–
	*P. aeruginosa*	63 (70)	48 (75)	18 (78)	14 (79)	126 (12)	43 (42)	115 (24)	36 (56)	54 (20)	–	–
	*S. aureus*	171 (73)	192 (71)	166 (80)	66 (59)	56 (43)	119 (61)	169 (53)	37 (68)	11 (73)	–	–
Diarrhea	*E. coli*	348 (75)	348 (83)	47(11)	541(47)	348 (6)	108(6)	348(30)	348(26)	240(9)	–	–
	*Salmonella Spp*	116 (16)	102(6)	39 (74)	155 (12)	234 (8)	195 (5)	214 (8)	234 (13)	70 (3)	91 (51)	–
	*Shigella Spp*	127 (51)	103 (85)	99 (83)	66 (20)	129 (13)	58(16)		158 (44)	122 (5)	–	–
	*Campylobacter*	102 (53)	119 (57)	20 (80)	44 (36)	119 (14)	64 (16)	102 (26)	102 (17)	82 (12)	–	–
Surgical site Infection	*S. aureus*	171 (73)	192 (71)	266 (80)	66 (59)	56 (43)	119 (61)	169 (53)	37 (68)	11 (72)	80 (85)	22 (9)
	*E. coli*	60 (73)	90 (90)	35 (74)	61(97)	90 (43)	90 (62)	84 (55)	64 (39)	9 (44)	–	12 (0)
	*K. pneumoniae*	44 (80)	83 (96)	43 (100)	69 (84)	83 (33)	83 (70)	83 (47)	40 (60)	–	–	–
	*P. aeruginosa*	15 (53)	32 (100)	17 (100)	26 (100)	32 (59)	32 (94)	21 (52)	20 (80)	5 (20)	20 (55)	–
Sepsis	*S. aureus*	98 (47)	91 (58)	–	20 (0)	98 (18)	82 (23)	98 (33)	91 (25)	–	–	–
	*E. coli*	47 (60)	31 (68)	–	29 (41)	47 (21)	29 (38)	47 (83)	31 (32)	–	–	–
	*K. pneumoniae*	19 (42)	19 (94)	–	19 (32)	19 (16)	19 (79)	19 (16)	31.58	–	–	–
Pneumonia	*S. pneumoniae*	77 (58)	60 (45)	–	–		60 (8)	1 (0)	77 (6)	–	–	–
	*K. pneumoniae*	29 (45)	3 (100)	3 (100)	33 (9)	29 (7)	59 (54)	59 (64)	29 (17)	–	–	–
	*S. aureus*	42 (52)	24 (92)	–	–	18 (11)	24 (88)	18 (17)	42 (17)	–	–	–
	*E. coli*	19 (32)	10 (90)	3 (0)	13 (15)	26 (8)	29 (38)	29 (31)	19 (0)	–	–	–
Middle ear infections	*S. aureus*	–	–	–	46 (61)	–	–	–	54 (26)	–	55 (35)	–
	*Proteus spp.*	227 (63)	227 (75)	–	119 (78)	–	–	283 (64)	227 (37)	227 (31)	–	–
	*P. aeruginosa*		88 (90)	–	28 (89)	–	–	–	18 (78)	–	–	–
	*Klebsiella spp.*	89 (66)	51 (82)	–	–	–	–	–	43 (51)	30 (40)	–	–
	*E. coli*	–	7 (86)	6 (50)	–	–	–	–	7.1	–	–	–
Nosocomial infections	*S. aureus*	77 (35)	–	77 (51)	77 (23)	87 (21)	87 (18)	87 (16)	87 (24)	–	–	–
	*E. coli*	67 (33)	–	33 (64)	33 (55)	42 (31)	42 (38)	42 (33)	42 (36)	–	–	–
	Citrobacter spp.	48 (29)	–	29 (76)	29 (55)	33 (12)	33 (45)	33 (40)	33 (33)	–	–	–
	Klebsiella spp.	47 (15)	–	15 (87)	15 (67)	15 (27)	15 (47)	15 (33)	15 (27)	–	–	–

The first number indicates the total number of resistant isolates for the specific pathogen and medication, and the number in the bracket is percent of resistant isolates

TSX, trimethoprim-sulfamethoxazole; AMP, ampicillin; AMC, amoxicillin; AMC-CIA, amoxicillin-clavulanic acid; CIP, ciprofloxacin; CRO, ceftriaxone; GCN, gentamicin; CLO, chloramphenicol; NOR, norfloxacin; OX/MET, oxacillin and or methicillin; CARBS, carbapenems (imipenem and or meropenem)

**Table 5:** Disease/clinical condition-based resistance pattern obtained from the current systematic review and comparison with existing Guidelines

Disease	Top pathogens by disease in their order of frequency	STG (drugs recommended [≥ 1 drug(s)]		Resistance n (%)		UpToDate V. 21.6		Resistance n (%)	
		Preferred	Alternative	Preferred	Alternative	Preferred	Alternative	Preferred	Alternative
Urinary Tract Infection (UTI)	*E. coli* *K. pneumoniae* *P. aeruginosa* *S. aureus* *Proteus spp.*	Ciprofloxacin Norfloxacin	Nitrofurantoin Cefpodoxime proxetil Trimethoprim-Sulfamethoxazole Ceftriaxone	518(30) 207(31)	262(18) 123(37) 1038(57) 501(36)	Nitrofurantoin Trimethoprim-Sulfamethoxazole Fosfomycin Ciprofloxacin Ceftriaxone	Pivmecillinam	262 (18) 1038(57) 518 (30) 681 (32)	No data
Diarrhea	*S. aureus* *Klebsiella spp.* *Campylobacter* *Salmonella* *Shigella*	Ciprofloxacin	Sulfamethoxazole-trimethoprim, Ceftriaxone	70 (10)	388 (57) 34 (9)	Ciprofloxacin Trimethoprim-Sulfamethoxazole Azithromycin Ceftriaxone	Ampicillin Amoxicillin	70 (10) 388(57) 10 (34) 34 (9)	115 (60) 476 (71)
Surgical Site Infection (SSI)	*S. aureus* *E. coli* *K. pneumoniae* *CoNS* *P. aeruginosa*	Cefazolin Ciprofloxacin	Cefuroxime Metronidazole Penicillin G	6166(45)7 (92)	60 (85) No data 139(67)	Cefazolin Cefuroxime Metronidazole Ampicillin-sub lactam	Vancomycin Gentamicin Clindamycin Ciprofloxacin Levofloxacin Aztreonam	6166(45) 60 (85) No data No data	1 (1) 253 (52) 64 (36) 166 (45) No data No data
Pneumonia	*S. pneumoniae* *K. pneumoniae* *S. aureus* *E. coli* *H. influenza*	Clarithromycin Amoxicillin	Azithromycin Doxycycline Amoxicillin/clavulanic acid	No data 14 (88)	No data 7 (8) 34 (40)	Amoxicillin		14 (88)	
Severe pneumonia		Ceftriaxone Penicillin G Azithromycin Clarithromycin		110 (46) 38 (32) No data No data		Ampicillin Gentamicin	Ceftriaxone	112 (90) 75 (42)	110 (46)
Hospital Acquired pneumonia		Ceftazidime Vancomycin Imipenem Meropenem	Gentamicin Ciprofloxacin Ceftriaxone	56 (58) No data No data No data	75 (42) 37 (27)	Vancomycin Linezolid Ticarcillin Piperacillin Tigecycline Telavancin Naficillin Oxacillin Imipenem-cilastatin, Ertapenem Meropenem doripenem	No data No data No data No data		
Aspiration pneumonia		Metronidazole Ceftriaxone Clindamycin	Amoxicillin/clavulanic acid	No data 47 (33)		Penicillin Metronidazole Amoxycillin	Levofloxacin Macrolides Cephalosporin	No data No data	No data No data
Sepsis (neonatal)		Ampicillin Gentamicin	Penicillin G Gentamicin	184 (68) 155 (52)	155 (52)	Ampicillin Gentamicin	Ceftriaxone	184 (68) 155 (52)	76 (35)
Ocular infections (Bacterial conjunctivitis And neonatal conjunctivitis)	*S. aureus* *CoNs* *E. coli* *S. pyogenes* *S. pneumoniae*	Chloramphenicol Crystalline Pencil line	Tetracycline Gentamicin Ciprofloxacin Tobramycin Ceftriaxone Cefotaxime	154 (28)	271 (48) 125 (26) 109 (17) No data	Erythromycin Trimethoprim	Bacitracin Sulfacetamide Polymyxin-bacitracin Fluoroquinolone Azithromycin	182 (39) 162 (33)	No data No data 109 (17) No data
Vaginal infections (discharge	*S. aureus* *E. coli* *S. pneumoniae* *K. pneumoniae* *Enterococci*	Ceftriaxone Doxycycline Metronidazole	Clindamycin Erythromycin Azithromycin Tinidazole Ciprofloxacin	27 (31) No data No data No data	58 (33) 95 (46) No data No data 69 (25)	Azithromycin Doxycycline Ceftriaxone Clindamycin Metronidazole	Ofloxacin Levofloxacin Spectinomycin Tinidazole Secnidazole	No data No data 27 (31) 58 (33) No data	No data No data No data No data No data
Middle ear infections	*S. aureus* *Proteus spp.* *P. aeruginosa* *Klebsiella spp.* *E. coli*	Amoxicillin	Ampicillin Amoxicillin/Clavulanate Ciprofloxacin Chloramphenicol Ceframed	584 (74)	1077 (77) 433 (66) 197 (14) 701 (43) No data	Amoxicillin	Cefdinir Cefpodoxime Cefuroxime Ceftriaxone Trimethoprim-Sulfamethoxazole	584 (74)	No data No data 1425 (70) 1148 (54)

n = overall count of resistant bacterial isolates

*Campylobacter*, *Shigella* and *Salmonella* has been reported as common causes of hemorrhagic diarrhea. 9–13% of those organisms were resistant to ciprofloxacin, the preferred regimen in the Ethiopian STG. Resistance to TMP/SMX was high at 30–49% and 10–23% of the strains were resistant to ceftriaxone, the IV alternate regimen (Tables 4,5).

The most common bacterial etiologies in surgical site infections (SSIs) were *S. aureus, E. coli, K. pneumoniae*, and *P. aeruginosa* (Table 4). Comparing Ethiopian STG and our AMR data, 45% of *S. aureus* and around 20% of the Gram-negative organisms were resistant to cefazoline and ciprofloxacin, the first line surgical site infections (SSIs) prophylaxis antimicrobials respectively (Table 5). *S. pneumoniae, K. pneumoniae, S. aureus, E. coli* and *H. influenzae* were identified as the top five causative agents of community acquired pneumonia. Among these amoxicillin, ceftriaxone and penicillin G showed 88%, 46% and 32% resistance, respectively. There was no susceptibility data available for other drugs recommended in national STG for the management of pneumonia.

In the Ethiopian national STG, ampicillin and gentamicin are described as preferred antimicrobial agents for treating sepsis while penicillin G and gentamicin are the alternatives. All the causative agents of sepsis (Tables 4, 5) showed high levels of resistance to ampicillin (68%), gentamicin (52%), and ceftriaxone (35%).

The top five causative agents for ocular infections were *S. aureus*, coagulase-negative staphylococci (CoNS), *E. coli*, *S. pyogenes* and *S. pneumoniae* (Table 5). Among the preferred antimicrobial agents, chloramphenicol showed a resistance rate of 28%. The alternatives had varying degrees of resistance with the highest for tetracycline (48%) and the lowest for ciprofloxacin (17%).

The top five causative agents of middle ear infection were *S. aureus*, *Proteus* spp, *P. aeruginosa*, *Klebsiella* spp. and *E. coli* in descending order of frequency. The preferred antimicrobial agent to treat middle ear infection is amoxicillin. The overall rate of resistance to amoxicillin is 74%. Based on the national STG the alternative antimicrobial agents are ampicillin, amoxicillin/clavulanate, ciprofloxacin and chloramphenicol. Among the alternative antimicrobials ciprofloxacin demonstrated lower resistance rate (14%), while the remaining showed a high resistance rate (Table 5).

## Discussion

Determining the rates of antimicrobial resistance and generating evidence is an important step in improving treatment outcomes and designing an appropriate intervention strategy to mitigate emergence and spread of resistant bacterial strains[18]. In this review, 131 studies reported antibiotic resistance across different parts of Ethiopia for commonly used antibiotics and met quality standards for inclusion. Most of the studies were hospital-based with a primary sample source of urine, ear/nose/throat, and stool. Over two-thirds of the papers were disease-specific, mainly urinary tract infection, diarrhea, and wound infections. Highly studied pathogens were *S. aureus* and *E. coli.* Antimicrobial susceptibility testing was conducted on 73 antimicrobial agents, the commonest ones being ciprofloxacin, gentamicin, trimethoprim-sulfamethoxazole, tetracycline, chloramphenicol, ampicillin, ciprofloxacin, ceftriaxone, doxycycline, and erythromycin. Prior studies have reported that AMR prevalence in Ethiopia is increasing at an alarming rate. In our current review, pathogens causing diverse disease conditions showed 30–85% resistance to key antimicrobial agents described in the Ethiopian Standard Treatment Guideline (STG). These findings are likely to challenge existing empirical antibiotic treatment strategies given the growing prevalence of AMR in the country.

We highlighted the resistance patterns of pathogens commonly implicated in various clinical conditions including urinary tract infection, diarrhea, surgical site infection, pneumonia, ocular, and middle ear infections. The most frequently isolated pathogens from the above-mentioned clinical diseases were *E. coli, S. aureus, K. pneumoniae* and *P. aeruginosa* (Table 5) [19]*.* The high rate of methicillin-resistant *Staphylococcus aureus* ((MRSA) reported in Ethiopia is similar with reports from other African countries. A review by Zigmond et al. [20] showed the average prevalence of MRSA in sub-Saharan and central Africa was 40.4% and in Northern African countries it was 48.6%. In Botswana, MRSA prevalence ranges from 23 to 44% [21]. In this study, vancomycin resistance varied from 8 to 20%. This agrees with the data from East (17.9%) and North (15.9%) Africa; however, it contrasts with the data from South African 74.8% and West 2.8% regions.

In our study, *S. pyogenes* resistance to penicillin, ceftriaxone, and amoxicillin/clavulanic acid was 24%, 26% and 32%, respectively. In other African countries, *Streptococci* species showed higher rate of resistance (40%) to ceftriaxone [22]. However, a report in Tanzania indicated a resistance rate to ceftriaxone (4.4%), penicillin (38.3%) and amoxicillin/clavulanic acid (40.4%) [23]. The possible explanation for these discrepancies might be due to differences in the study population, methods used, geographical variations in disease burden, practice of antimicrobials use and presence, and implementation of national antimicrobials use policy. Most health care facilities in Ethiopia do not have microbiology laboratories for diagnosis and sensitivity. Even at the centers where this is available, microbiological results are usually available after 24–72 h, and thus, early treatment for infections is often empirical, guided by the clinical presentations [24]. Nevertheless, our review revealed that most of the empiric first-line and alternative antibiotics recommended by the Ethiopia STG showed resistance rates of more than 20%. The accepted limit of resistance where an antimicrobial agent should no longer be used for empirical treatment is usually around 20% [25]. In the current review, first-line drugs (ciprofloxacin and norfloxacin) for the treatment of uropathogens have shown similar rate of resistance (30%). Nitrofurantoin was the only drug with a relatively low resistance profile but this is only recommended for cystitis and not complicated UTIs. On the other hand, pathogens causing bacterial dysentery would better treat with ciprofloxacin and ceftriaxone (9%), whereas trimethoprim-sulfamethoxazole showed a higher resistance (57%) to both urinary and gut pathogens. The other striking finding was that pathogens commonly causing surgical site infections were not well covered by the recommended prophylactic drugs cefazolin, ciprofloxacin, and cefuroxime.

The management of common community-acquired pneumonia with amoxicillin and amoxicillin-clavulanic acid [26] seems as well to be challenged in the face of 87% resistance. Though clarithromycin and azithromycin are recommended by the Ethiopian STG to treat community acquired pneumonia in adults, only limited data has been available on sensitivity of pathogens to these drugs. Similarly, resistance to drugs used for management of severe pneumonia, ceftriaxone and penicillin G, were found to be above 30% which is quite concerning [17].

Similarly, the two first line empiric treatment drugs for neonatal sepsis (ampicillin and gentamicin) [27] may not be effective due to high resistance to both drugs. Middle ear infection may be better treated with ciprofloxacin which showed a lower rate of resistance (14%) in comparison to amoxicillin, amoxicillin-clavulanic acid, and chloramphenicol. A similarly high rate of resistance to the common pathogens was reported by antimicrobial resistance review in Africa [3] and this needs due attention. Even though the reported disease entities in this review are not exhaustive, the recommended and the alternative antimicrobials for the empiric treatment of common infections in the Ethiopian STG have shown a high degree of resistance. Our findings therefore, suggest that a review of the existing STG is desirable. Moreover, the list of drugs in the national STG are few and most of the newer antimicrobials described in the international recommendations are not included in the list. Hence, it is also important to expand treatment options by incorporating newer and more effective antimicrobials in the essential drug list.

### Clinical implications

The outcome of our current review has critical implications to clinical practice and policy framework. In this study, most of the preferred antibiotics recommended by the Ethiopia STG showed a high level of resistance. This exposes significant barriers to effective empiric treatment for diseases such as urinary tract infections, community acquired pneumonia and surgical site infections.

Resistance is not only limited to the preferred drug regimens but also found in alternative antimicrobials. However, due to limited availability of culture facilities particularly in low- and middle-income countries, most clinicians prescribe empirical treatment based on national and international guidelines [28]. Unless national treatment guidelines are revised regularly based on local evidence, clinicians are likely to prescribe antimicrobials which might be ineffective, jeopardizing lives and community risk. Therefore, it is our strong recommendation that Ethiopia revise the national treatment guidelines using the best available evidence. In addition, it is beneficial to significantly reduce the practice of empiric treatment by expanding diagnostic facilities throughout the country, allowing patient treatment to be tailored to etiologic diagnosis and sensitivity results.

### Strength and limitations

To the best of our knowledge, this is the first comprehensive analysis of AMR data generated in Ethiopia. Our search was limited to a ten-year period (2009–2019). This helped us to focus on studies that reflect the recent AMR patterns in Ethiopia. The results should be interpreted with caution as the reviewed studies were highly heterogeneous reporting a wide range of pathogens, antimicrobial agents, disease conditions, and studies were conducted mainly in hospitals, performed on different types of specimens and sources, and with different sample sizes. In addition, the disease entities described in the included studies were not comprehensive enough to compile antibiotic resistance profiles in all infectious diseases of public health importance.

In addition, since the majority of the reviewed studies did not document antibiograms, we were unable to extract data relevant to multidrug resistance. Due to the heterogeneous nature of the studies, we preferred to make our data presentation more descriptive. The other limitation was that we used only PubMed/Medline. However, this would have only minimally affected our findings, as we already included a large number of studies conducted in Ethiopia.

## Conclusion

In conclusion, the challenge faced by the medical practice due to antimicrobial resistance is immense and complex [29]. One of the reasons why the impact of AMR is overlooked in hospital settings could be the extensive practice of empirical treatment in our health system, which is worsened by the lack of a system that evaluates treatment outcomes of patients. We could not find any study conducted in Ethiopia that evaluated the impact of antimicrobial resistance and empirical antibiotics treatment on morbidity and mortality outcomes.

This review revealed that common bacterial isolates (*S. aureus, E. coli, K. pneumoniae* and *P. aeruginosa)* are resistant to the commonly used antibiotics (beta-lactams including third generation cephalosporins) [30]. In addition, the recommended and the alternative antimicrobial agents for the empiric treatment of common infections (urinary tract infections, sepsis, pneumonia and diarrhea) in the Ethiopian STG have high degree of resistance. To mitigate the problem, we would propose the following recommendations: revise the existing national antimicrobial standard treatment guidelines, develop treatment guidelines for appropriate use of antibiotic agents in health facilities, improve laboratory infrastructure for culture and drug susceptibility testing, establish/strengthen antimicrobial stewardship programs, increase uptake of research evidence to positively influence clinical practices (e.g., empirical treatment) and policy framework, and promote rational use of antimicrobial agents through community awareness. We also recommend conducting a prospective study that examines the prevalence of AMR and its impact on patient outcomes. Furthermore, a broader research focusing on determinant factors promoting the development and spread of antimicrobial resistance in the country should be conducted.

## Supplementary Information

**Additional file 1.** A detail account of all articles included in the review.

## Data Availability

The datasets used in this review are available upon request of the corresponding author. All relevant data are given separately as a supplementary data/material.

## Abbreviations

AMR: Antimicrobial resistance
CoNS: Coagulase negative staphylococci
ESBL: Extended—spectrum beta lactamase
MRSA: Methicillin resistant *Staphylococcus aureus*
PRISMA: Preferred reporting items for systematic reviews and meta-analyses
SD: Standard deviation
SSA: Sub-Saharan Africa
SSI: Surgical site infection
STG: Standard treatment guideline
TMS: Trimethoprim-sulfamethoxazole
USD: United States Dollar
UTI: Urinary tract infection
WHO: World Health Organization

## References

[1] Kimang’a AN (2012). A situational analysis of antimicrobial drug resistance in Africa: are we losing the battle?. Ethiop J Health Sci.

[2] Jasovský D, Littmann J, Zorzet A, Cars O (2016). Antimicrobial resistance-a threat to the world’s sustainable development. UPS J Med Sci.

[3] Worku F, Tewahido D (2018). Retrospective assessment of antibiotics prescribing at public primary healthcare facilities in Addis Ababa. Ethiopia Interdiscip Perspect Infect Dis.

[4] WHO. The evolving threat of antimicrobial resistance: options for action. World Health Organization; 2012.

[5] Okeke IN, Aboderin OA, Byarugaba DK, Ojo KK, Opintan JA (2007). Growing problem of multidrug-resistant enteric pathogens in Africa. Emerg Infect Dis.

[6] Zenebe T, Kannan S, Yilma D, Beyene G (2011). Original Article invasive bacterial pathogens and their antibiotic susceptibility patterns in Jimma Specialized Hospital. Ethiop J Health Sci.

[7] Newton PN, Green MD, Mildenhall DC, Plançon A, Nettey H, Nyadong L (2011). Poor quality vital anti-malarials in Africa—an urgent neglected public health priority. Malaria J.

[8] Ndihokubwayo JB, Yahaya AA, Desta AT, Ki-Zerbo G, Odei EA, Keita B (2013). Antimicrobial resistance in the African Region: issues, challenges and actions proposed. Afr Heal Monit.

[9] Cockburn R, Newton PN, Agyarko EK, Akunyili D, White NJ (2005). The global threat of counterfeit drugs: why industry and governments must communicate the dangers. PLoS Med.

[10] Murray CJL, Ezzati M, Flaxman AD, Lim S, Lozano R, Michaud C (2012). GBD 2010: a multi-investigator collaboration for global comparative descriptive epidemiology. Lancet (Lond Engl).

[11] Ampaire L, Muhindo A, Orikiriza P, Mwanga-Amumpaire J, Bebell L, Boum Y (2016). A review of antimicrobial resistance in East Africa. Afr J Lab Med.

[12] Wikler MA, Cockerill FR, Craig WA, Dudley MN, Eliopoulos GM, Hecht DW, et al. M02-A12: performance standards for antimicrobial disk susceptibility tests; Approved Standard Twelfth Edition, vol. M0-A12. 2015.

[13] Moges F, Endris M, Mulu A, Tessema B, Belyhun Y, Shiferaw Y (2014). The growing challenges of antibacterial drug resistance in Ethiopia. J Glob Antimicrob Resist.

[14] Administration D, of Ethiopia CA. Antimicrobials use, resistance and containment baseline survey syntheses of findings. Drug Administration and Control Authority of Ethiopia Addis Ababa, Ethiopia; 2009.

[15] Moher D, Liberati A, Tetzlaff J, Altman DG, Grp P (2009). Preferred reporting items for systematic reviews and meta-analyses: the PRISMA statement (reprinted from annals of internal medicine). Phys Ther.

[16] Modesti PA, Reboldi G, Cappuccio FP, Agyemang C, Remuzzi G, Rapi S (2016). Panethnic differences in blood pressure in Europe: a systematic review and meta-analysis. PLoS ONE.

[17] EFDA. Standard Treatment Guidelines for General Hospitals, Ethiopia. Third Edition: Ethiopian Food and Drug Authority. 2017.

[18] Lee CR, Cho IH, Jeong BC, Lee SH (2013). Strategies to minimize antibiotic resistance. Int J Environ Res Public Health.

[19] Birhanu Y, Endalamaw A (2020). Surgical site infection and pathogens in Ethiopia: a systematic review and meta-analysis. Patient Saf Surg.

[20] Zigmond J, Pecan L, Hájek P, Raghubir N, Omrani AS (2014). MRSA infection and colonization rates in Africa and Middle East: a systematic review and meta-analysis. Int J Infect Dis.

[21] Falagas ME, Karageorgopoulos DE, Leptidis J, Korbila IP. MRSA in Africa: Filling the Global Map of Antimicrobial Resistance. PLoS ONE. 2013;8. 10.1371/journal.pone.0068024 PMC3726677 23922652

[22] Bashir A, Garba I, Aliero AA, Kibiya A, Abubakar MH, Ntulume I (2019). Superbugs-related prolonged admissions in three tertiary hospitals, Kano State. Nigeria Pan Afr Med J.

[23] Abraham ZS, Tarimo O, Kahinga AA, Ntunaguzi D, Mapondella KB, Massawe ER (2019). Prevalence and clinical characteristics of otitis externa among patients attending Otorhinolaryngology Department at Muhimbili National Hospital, Tanzania. Int J Otorhinolaryngol Head Neck Surg.

[24] Wozniak TM, Paterson D, Halton K (2017). Review of the epidemiological data regarding antimicrobial resistance in Gram-negative bacteria in Australia. Infect Dis Heal.

[25] Jakobsson T, Forsum U, Bean DC, Krahe D, Wareham DW (2008). Antimicrobial resistance in community and nosocomial *Escherichia coli* urinary tract isolates. London.

[26] Kaysin A, Viera AJ (2016). Community-acquired pneumonia in adults: diagnosis and management. Am Fam Phys.

[27] Bibi S, Chisti MJ, Akram F, Pietroni MAC (2012). Ampicillin and gentamicin are a useful first-line combination for the management of sepsis in under-five children at an urban hospital in Bangladesh. J Health Popul Nutr.

[28] Sulis G, Adam P, Nafade V, Gore G, Daniels B, Daftary A (2020). Antibiotic prescription practices in primary care in low-and middle-income countries: A systematic review and meta-analysis. PLoS Med.

[29] Sosa A de J, Byarugaba DK, Amábile-Cuevas CF, Hsueh P-R, Kariuki S, Okeke IN. Antimicrobial resistance in developing countries. Springer; 2010.

[30] Tesera H, Derbie A, Mekonnen D. Bacterial isolates and their antimicrobial resistance profile among patients presumptive for meningitis at a referral hospital, northwest Ethiopia. Ethiop J Heal Dev. 2020;34.

