# Supplementary material for: Prevalence of antimicrobial resistance and its clinical implications in Ethiopia: a systematic review
Source: Antimicrob Resist Infect Control. 2021 Dec 3;10:168. doi: 10.1186/s13756-021-00965-0 (PMC8642948; doi:10.1186/s13756-021-00965-0)
Supplement: Supplementary file 1 — Additional file 1. A detail account of all articles included in the review. [file 13756_2021_965_MOESM1_ESM.docx]

Additional file 1. A detail account of all articles included in the review

| **Author/ Publication year (ref)** | **Sample size** | **Sample type** | **Study Region** | **Setup** | **Clinical condition/ disease** | **Clinical site** | **Quality score** | **Isolate (n)** | |
| --- | --- | --- | --- | --- | --- | --- | --- | --- | --- |
| **Urinary Tract Infection (UTI)** |  |  |  |  |  |  |  |  | |
| Alemu *et al* 2012 [1] | 385 | Urine | Amhara | H | UTI |  | 7 | 40 | |
| Ayelign *et al* 2018 [2] | 310 | Urine | Amhara | H | UTI | Pedi clinic | 7 | 82 | |
| Bekele et al 2015 [3] | 73 | Urine | Oromia | H | Catheterized |  | 6 | 36 | |
| Eshetie et al 2015 [4] | 442 | Urine | Amhara | H | UTI | Out and in-patient | 7 | 183 | |
| ^6^Ferede et al 2018 [5] | 422 | Urine, blood, body fluid, CSF and Pus | AA | H | UTI  Septicemia  Wound infection  Endocarditis  Meningitis | NA | 7 | 15 | |
| Kibret M. and Abera, B 2011 [6] | 3149 | Ear discharge/swab  Ophthalmic Urine  Wound swab | Amhara | LBD | UTI, Ear infection |  | 8 | 446 | |
| Mamuye, Y. 2016 [7] | 424 | Urine | AA | H | UTI |  | 8 | 95 | |
| Nigussie et al 2017 [8] | 240 | Urine | SNNPR | H | UTI | diabetic clinic | 6 | 33 | |
| Yeshitela et al 2012 [9] | 413 | Urine | AA | H | UTI, septicemia, wound infection, endocarditis and meningitis |  | 9 | 46 | |
| Duffa et al 2018 [10] | 384 | Urine | AA | H | UTI | Pedi clinks/ward | 10 | 61 | |
| Beyene et al. 2011 [11] | 228 | Urine | Oromia | H | UTI |  | 6 | | 21 |
| Demilie et al 2012 [12] | 367 | Urine | Amhara | H | UTI/Pregnant |  | 6 | | 35 |
| Derese et al 2016 [13] |  | Urine | Diredawa | H | UTI | ANC clinic | 8 | | 26 |
| Woldemariam et al 2019 [14] | 248 | Urine | AA | H | UTI | Endocrine clinic | 7 | |  |
| Wondimeneh et al 2014 [15] | 53 | Urine | Amhara | H | UTI | Obstetric fistula wards | 8 | | 28 |
| Tuem et al 2019 [16] | 1080 | Urine | Tigray | LBD | UTI |  | 8 | | 308 |
| Gessese et al 2917 [17] | 300 | Urine | Oromia | H | UTI |  | 8 | | 56 |
| Bitew et al 2017 [18] | 712 | Urine | AA | LBD | UTI | Medical laboratory (private) | 8 | | 256 |
| Kibret et al 2014 [19] | 1404 | Urine | Amhara | LBD | UTI | Regional Lab | 7 | | 319 |
| Awoke et al 2019 [20] | 143 | Urine | Oromia | H | Urinary catheterized | Gyn / Obs, surgical, and medical wards | 5 | | 60 |
| Gutema et al 2018 [21] | 233 | Urine | Oromia | H | UTI on DM | Diabetic clinic | 10 | | 39 |
| Abejew et al 2014 [22] | 2486 | Urine; Urethral discharge; Vaginal Discharge | Amhara | LBD | UTI | Data base | 8 | | 680 |
| Marami et al 2019 [23] | 350 | Urine | Harrer | H | UTI | Hospital ART clinic | 10 | | 63 |
| Merga et al 2018 [10] |  |  |  |  |  |  |  | |  |
| Mitiku *et al* 2018 [24] | 269 | Urine | SNNP | H | UTI | Pediatrics OPD | 9 | | 71 |
| Gashe et al 2018 [25] | 248 | Open wound discharge; Sputum; Stool; Urine | Oromia | H | UTI, open wounds, pneumonia, and upper RTI |  | 6 | | 248 |
| **Diarrhea** |  |  |  |  |  |  |  | |  |
| Abebe et al 2018 [26] | 204 | Stool | SNNPR | H | Diarrhea | Pediatrics care | 6 | |  |
| Adugna et al 2015 [27] | 422 | Stool | Amhara | HC | Diarrhea | Pedi clinic | 8 | | 204 |
| Alemu et al 2019 [28] | 272 | Stool | Amhara | HC  H | Diarrhea | Pedi clinic | 9 | | 29 |
| Eguale et a1 2015 [29] | 957 | Stool | AA | HC  H | Diarrhea |  | 8 | | 67 |
| Gebreegziabher et al 2018 [30] | 260 | Stool | Tigray | HC  H | Diarrhea |  | 5 | | 37 |
| Gebrekidan et al 2015 [31] | 216 | Stool | Tigray | H | Diarrhea |  | 8 | | 15 |
| GebreSilasie et al 2018 [32] | 253 | Stool | AA | HC  H | Diarrhea |  | 9 | | 61 |
| Lamboro et al 2016 [33] | 176 | Stool | Oromia | H | Diarrhea | Outpatient | 9 | | 21 |
| Getamesay et al 2014 [34] | 158 | Stool | SNNPR | HC  H | Diarrhea | not specified | 5 | | 35 |
| Terfassa, et al 2018 [35] | 422 | Stool | Oromia | H | Diarrhea | OPD | 8 | | 273 |
| Getachew et al 2011[36] | 215 | Stool | Amhara | H | Diarrhea | Not specific | 7 | | 32 |
| Ewnetu et al 2010 [37] | 164 | Stool | Amhara | C | Diarrhea Stomach cramps, Fever | C | 6 | | 17 |
| Lengerh et al 2013 [38] | 285 | Stool | Amhara | H | Diarrhea | Pediatric ward | 8 | | 44 |
| Tafa et al 2014 [39] | 228 | Stool | Oromia | H | Diarrhea | Pediatrics OPD and wards | 7 | | 38 |
| Teshome et al 2019 [40] | 232 | Stool | Oromia | HC  H | Diarrhea | Wards and OPD | 10 | | 42 |
| Zelelie et al 2019 [41] | 163 | Stool | Amhara | H | Diarrhea | Pedi OPD/Ward | 7 | | 91 |
| Eguale et al 2018 [42] | 68 | Stool | AA | HC  H | Diarrhea | Not specific | 6 | | 68 |
| Abera et al 2010 [43] | 81 | Stool | Amhara | C | *Cholera* | Not specific | 4 | | 81 |
| **Wound infection** |  |  |  |  |  |  |  | |  |
| Mohammed et al 2017 [44] | 137 | wound swab | Amhara | H | Wound infection | OPD/IPD | 7 | | 115 |
| Mulualem, et al 2012 [45] | 359 | Open wound discharge; Sputum; Stool; Urine, Other; | Oromia | H | Wound infection | OPD & IPD | 7 | | 67 |
| Mama et al 2019 [46] | 50 | wound swab | SNNPR | H | Wound infection |  | 7 | | 36 |
| Sewunet et al 2013 [47] | 50 | Blood; wound swab | AA | HH | Wound infection /Burn | Burn Center | 4 | | 68 |
| Azene et al 2011[48] | 599 | Open wound discharge | Amhara | LDB | Wound infection | Regional lab | 9 | | 500 |
| Godebo et al 2013 [49] | 322 | Burn; open wound discharge | Oromia | H |  | Inpatient and OPD | 6 | | 384 |
| Mama et al 2014 [50] | 150 | wound swab | Oromia;  SNNPR | H | Wound infection |  | 5 | | 145 |
| **Ear infection** |  |  |  |  |  |  |  | |  |
| Hailegiyorgis et al 2018 [51] | 196 | Ear discharge/swab | AA | HC  H | Otitis media | Pediatrics OPD | 7 | | 101 |
| Deyno et al 2017 [52] | 117 | Ear discharge/swab | SNNPR | H | ENT | ENT clinic | 5 | | 33 |
| Hailu, et al 2016 [53] | 368 | Ear discharge/swab | Amhara | LBD | Ear infection/otitis media | Regional lab database | 6 | | 289 |
| Muluye et al 2014 [54] | 102 | Ophthalmic | Amhara | H, LDB | Ocular infection | microbiology lab register | 8 | | 62 |
| Gorems et al 2018 [55] | 173 | Ear discharge/swab | Oromia | H  HC | Ear infection | ENT | 9 | | 179 |
| Wasihun et al 2015 [56] | 162 | Ear discharge | Tigray | H | Otitis media | ENT clinic | 7 | | 216 |
| Argaw-Denboba et al 2016 [57] | 1225 | Ear discharge | Amhara | LBD | Middle ear infection |  | 9 | | 1024 |
|  |  |  |  |  |  |  |  | |  |
| Abera et al 2009 [58] | 777 | Ear discharge/swab | Amhara | LBD | Otitis media | LBD | 7 | | 608 |
| **Eye infection** |  |  |  |  |  |  |  | |  |
| Shiferaw et al 2015 [59] | 160 | Ophthalmic | Oromia | H | Ocular infections |  | 10 | |  |
| Assefa et al 2015 [60] | 51 | Other | Amhara | H | Dacryocystitis | Ophthalmology OPD | 8 | | 31 |
| Muluye et al 2013 [61] | 228 | Ear discharge | Amhara | H  LDB | Ear infection | OPD | 8 | | 204 |
| Belyhun et al 2018 [62] | 210 | Ophthalmic | Amhara | H | External ocular infections | Ophthalmology | 6 | | 131 |
| Teweldemedhin et al 2017 [63] | 270 | Ophthalmic | Tigray | H | Ophthalmic infections | Ophthalmic OPD/Ward | 6 | | 180 |
| Tamrat et al 2019 [64] | 70 |  | Oromia | H | Eye diseases | Ophthalmology wards and OPD | 9 | | 56 |
| Getahun et al 2017 [65] | 312 | Ophthalmic | Amhara | H | Ocular infection | Ophthalmic OPD | 9 | | 191 |
| **Surgical Site Infection** |  |  |  |  |  |  |  | |  |
| Dessie et al 2016 [66] | 107 | wound swab | AA | Hl | SSI | Surgical | 8 | | 104 |
| Mulu et al 2012 [67] | 294 | Blood; Open wound discharge | Amhara | H | Post-operative infections | surgical and Gyn ward | 8 | | 42 |
| Gashaw et al 2018 [68] | 197 | Blood; open wound discharge;  sputum; urine | Oromia | H | SSI/health care associated infection | Not specific | 9 | | 126 |
| Kifilie et al 2018 [69] | 107 | Open wound discharge | Amhara | H | CS/episiotomy infection (SSI) | Gyn/Obs | 7 | | 90 |
| Mengesha et al 2014 [70] | 128 | Open wound discharge | Tigray | H | SSI | Orthopedic surgical ward | 9 | | 123 |
| Kahsay et al 2014 [71] | 184 | wound swab | Amhara | H | SSI | Surgical and Gyn/Obs wards | 7 | | 73 |
| **Sepsis** |  |  |  |  |  |  |  | |  |
| Seboxa et al 2015 [72] | 292 | Blood | AA | H | Septicemia | Medical, and EMR, surgery, Gyn wards | 4 | | 38 |
| Alebachew et al 2016 [73] | 100 | Blood | Amhara | H | sepsis | ART Clinic | 7 | | 31 |
| Negussie et al 2015 [74] | 201 | Blood | AA | H | Sepsis | Pediatric OPD & IPD) | 7 | | 56 |
| Sorsa et al 2019 [75] | 303 | Blood | Oromia | Hl | Neonatal sepsis | neonatal ICU | 7 | | 88 |
| G/Eyesus et al 2017 [76] | 251 | Blood | Amhara | H | Sepsis | Pedi ward & NICU | 9 | | 120 |
| **Pneumonia** |  |  |  |  |  |  |  | |  |
| Adhanom et al 2019 [77] | 252 | Other | Tigray | HC  H | Pneumonia | ART clinic | 6 | | 110 |
| Negash et al 2019 [78] | 549 | Blood | AA | H | Community acquired pneumonia | Pedi emergency | 8 | | 31 |
| Temesgen et al 2019 [79] | 414 | Sputum | Amhara | H | Community acquired pneumonia | Medical ward/OPD | 9 | | 167 |
| **STI and genital area infection** |  |  |  |  |  |  |  | |  |
| Ali et al 2016 [80] | 186 | Urethral and end cervical swab | Gambela | Hl | STI | OPD | 6 | | 21 |
| Hailemariam et al 2013 [81] | 215 | Vaginal/cervical swab | SNNPR | H | Suspected STIs | Gyn OPD | 7 | | 11 |
| Yeshanew et al 2018 [82] | 120 | Urethral discharge; Vaginal discharge | Amhara | HC  H | STI/ Gonorrhea | OPD | 10 | | 25 |
| Mulu et al 2015 [83] | 409 | Vaginal/cervical swab | Amhara | H | Vaginal infection | Different site | 8 | | 49 |
| Bitew et al 2017 [84] | 210 | Vaginal discharge | AA | H | Virginal infection | Gyn/Obs | 8 | | 151 |
| **Gastroenteritis** |  |  |  |  |  |  |  | |  |
| Desta et al 2016 [85] | 267 | Anorectal Swab  Stool | AA | H | Gastroenteritis | OPD/Inpatients  Medical, surgical, Gyn/Obs Pedi and Neonatal | 8 | | 295 |
| Kebede et al 2017 [86] | 215 | Stool | SNNPR | H | Gastroenteritis | ART clinic | 8 | | 29 |
| **Other diseases** |  |  |  |  |  |  |  | |  |
| Abera et al 2014 [87] | 1413 |  | Amhara | H | Trachomatous trichiasis | Surgical (trachomatous trichiasis) | 6 | | 438 |
| Fentie et al 2018 [88] | 216 | Blood; Ear discharge/swab; Open wound discharge; Urine |  | H | Cancer | Oncology center | 9 | | 43 |
| Arega et al, 2017 [89] | 107 | Blood | AA | H | bloodstream infections | cancer clinic | 8 | | 71 |
| Arega et al 2018 [90] | 76 | Blood | AA | H | Febrile cancer | Cancer clinic | 8 | | 82 |
| Deksissa 2019 [91] | 372 | Blood; Stool | Oromia | H | Febrile patients | OPD/Inpatient and ART clinic | 7 | | 10 |
| Marks et al 2017 [92] | 847 | Blood | Oromia | HC | Typhoid Fever |  | 9 | | 3 |
| Zenebe et al 2011 [93] | 260 | Blood | Oromia | H | Fever | Medical wards | 7 | | 23 |
| Wasihun et al 2015 [94] | 514 | Blood | Tigray | H | Febrile patients | OPD | 8 | | 144 |
| Wasihun et al 2015 [95] | 502 | Blood | Tigray | HC  H | Typhoid fever | OPD | 6 | | 115 |
| Mitiku et al 2019 [96] | 170 | Blood | Diredawa; Harrer | H | HIV | ART clinic | 9 | | 17 |
| Lemma et al 2015 [97] | 400 | Body surface (skin); Nasal swab; Others | Amhara | H | HIV under 15 children | pediatrics HIV clinics | 6 | | 281 |
| Tolera et al 2018 [98] | 433 | Blood; Nasal swab; Open wound discharge; throat(pharyngeal) swab; Urine, Other | Harari | H | Nosocomial infections | All major wards | 8 | | 54 |
| Feleke et al 2018 [99] | 260 | Blood; Open wound discharge;  Ophthalmic; Stool; Urine; Others | Amhara | H | Nosocomial infections | Surgical, Pediatrics, Medical, & Gyn | 7 | | 216 |
| **No disease or not specific** | |  |  |  |  |  |  | |  |
| Anagaw et al 2013 [100] | 153 | Blood; CSF; Ear discharge; Nasal swab; Open wound discharge; Ophthalmic; throat swab; Urine Other | Amhara | H | On all clinical samples | OPD/Ward | 7 | | 153 |
| Assefa, et al 2013 [101] | 234 | Nasal swab | Amhara | H | Well and sick children | Pediatrics OPD | 7 | | 96 |
| Assefa, et al 2018 [102] | 281 | Vaginal/cervical swab | AA | HC  H | Pregnancy | ANC clinic | 8 | | 41 |
| Gizachew  et al 2019 [103] | 385 | Anorectal swab  Vaginal Discharge | Amhara | H |  | Labor ward | 9 | | 98 |
| Mengist et al 2017 [104] | 180 | Vaginal/cervical swab | Oromia | H | Virginal GBS carriage | Gyn/Obs | 10 | | 22 |
| Toru et al 2018 [105] | 403 | Blood, CSF, Urine Other | Oromia | H | Not specified | Pediatrics ward and OPD | 8 | | 22 |
| Abdissa et al 2011 [106] | 937 | Throat  sab | AA  Amhara  Dire Dawa |  | On healthy school children |  | 6 | | 167 |
| Tadesse et al 2018 [107] | 259 | Urine | Tigray | H | Pregnant women, asymptomatic | ANC or Gyn | 7 | | 55 |
| Weldegebreal et al 2019 [108] | 212 |  | Harari | H | On medical equipment | OPD | 10 | | 137 |
| Genet et al 2011 [109] | 108 | Other | Oromia | H | Air sample | OR | 8 | | 51 |
| Dilnessa et al 2016 [110] | 1360 | Blood; CSF; Ear discharge/swab; Nasal swab; Open wound discharge; Sputum; Stool throat(pharyngeal) swab; Urine; Urethral discharge; Vaginal discharge | AA | H | On clinical sample | On clinical sample | 5 | | 194 |
| Abera et al 2016 [111] | 274 | Blood; Ear discharge; Open wound discharge; Urine | Amhara | HC  H | Clinical and drinking water | Hospital | 8 | | 210 |
| Alemayehu et al 2017 [112] | 240 | Nasal swab | AA | C | Healthy school children | School | 7 | | 49 |
| Agzie et al 2019 [113] | 196 | Blood | Tigray | LBD |  | blood bank | 8 | | 18 |
| Dagnew et al 2012 [114] | 200 | Nasal swab | Amhara | C  H | Healthy food  handlers | Students cafeteria | 8 | | 41 |
| Kahsay et al 2018 [115] | 384 | Nasal swab | Tigray | C  H | On healthy cleaners | Janitors (medical and non-medical) | 7 | | 69 |
| Getachew et al 2018 [116] | 356 |  | Amhara | H | Air sample | Surgical, OR, Maternity, ICU, Orthopedics and Dialysis | 7 | | 190 |
| Birri et al 2013 [117] | 28 |  | SNNPR | C | Health infants | Undefined | 6 | | 150 |
| Engda et al 2018 [118] | 384 |  | Amhara | H | Sample from environment | Medical, surgical  Fistula clinical, Gyn/Obs,  Eye clinic | 5 | | 57 |
| Garedew-Kifelew et al 2014 [119] | 423 | Stool | Amhara | H | Healthy food handlers | Food handlers | 7 | | 13 |
| Gizachew et al 2018 [120] | 385 | Ear swab; Nasal swab; Umbilical swab | Amhara | H | Newborn GBS carriage | Maternity ward | 9 | | 81 |
| Wada et al 2019 [121] | 714 | Nasal swab | SNNPR | C | Healthy school children | Primary schools | 9 | | 311 |
| Zeynudin et al 2018 [122] | 224 | Other | Oromia | H | Clinical specimens | Unspecified | 9 | | 112 |
| Solomon et al 2017 [123] | 216 |  | SNNPR | H | Air sample | Air from delivery room, ICU, OR | 8 | | 67 |
| Wondimu et al 2013 [124] | 137 | Blood | Amhara | H |  | Blood bank | 8 | | 21 |
| Reta et al 2017 [125] | 400 | Nasal swab | Amhara | H | Health KG children | KG school | 8 | | 52 |
| Legese et al 2018 [126] | 242 | Nasal swab | Tigray | H | On healthy health workers | Medical, Surgical, Gyn, Lab. OPD and Pharmacy | 7 | | 29 |
| Shibabaw et al 2014 [127] | 118 | Nasal swab | Amhara | H | On healthy health workers |  | 8 | | 34 |
| Solomon et al 2017 [128] | 216 |  | SNNPR | H | Air sample | delivery, ICU, OR | 8 | |  |
| Mulu, W et al 2017 [129] | 575 | Blood; CSF; Ear discharge; Nasal swab; Open wound discharge; Stool; throat (pharyngeal) swab; Urine; Urethral discharge; Vaginal discharge | Amhara | H | LBD | LDB | 7 | | 280 |
| Tigabu et al 2018 [130] | 622 | Nasal swab | Amhara | C | Health school children | Schools | 6 | | 143 |
| Kejela 2013 [131] | 354 | Nasal swab | Oromia | C | Health school children & prisoners | School and prison | 8 | | 169 |

AA: Addis Ababa; HC: Health Center; H: Hospital; LBD: Laboratory Based Data; C: Community; OPD: Outpatient Department; OR: Operation Room; ICU: Intensive Care Unit; KG: Kindergarten; Gyn/Obs: Gynecology and obstetrics ; ENT: Ear, Nose and Throat; UTI: Urinary Tract Infection; CS: Cesarean section; SSI: Surgical Site Infection; RTI: Respiratory Tract infection; HIV: Human Immune-deficiency Virus; ART: Antiretroviral therapy; CSF: Cerebrospinal Fluid; GBS: Group B Streptococci; GIT: Gastro Intestinal Infection; DM: Diabetes Mellitus.

**References**

1. Alemu A, Moges F, Shiferaw Y, Tafess K, Kassu A, Anagaw B, et al. Bacterial profile and drug susceptibility pattern of urinary tract infection in pregnant women at University of Gondar Teaching Hospital, Northwest Ethiopia. BMC Res Notes. 2012;5.

2. Ayelign B, Abebe B, Shibeshi A, Meshesha S, Shibabaw T, Addis Z, et al. Bacterial isolates and their antimicrobial susceptibility patterns among pediatric patients with urinary tract infections. Turkish J Urol. 2018;44:62–9.

3. Bekele T, Tesfaye A, Sewunet T, Waktola HD. Pseudomonas aeruginosa isolates and their antimicrobial susceptibility pattern among catheterized patients at Jimma University Teaching Hospital, Jimma, Ethiopia. BMC Res Notes. BioMed Central; 2015;8:1–4.

4. Eshetie S, Unakal C, Gelaw A, Ayelign B, Endris M, Moges F. Multidrug resistant and carbapenemase producing Enterobacteriaceae among patients with urinary tract infection at referral Hospital, Northwest Ethiopia. Antimicrob Resist Infect Control. ???; 2015;4:1–8.

5. Ferede ZT, Tullu KD, Derese SG, Yeshanew AG. Prevalence and antimicrobial susceptibility pattern of Enterococcus species isolated from different clinical samples at Black Lion Specialized Teaching Hospital, Addis Ababa, Ethiopia. BMC Res Notes. BioMed Central; 2018;11:8–13.

6. Kibret M, Abera B. Antimicrobial susceptibility patterns of E. coli from clinical sources in northeast Ethiopia. Afr Health Sci. 2011;11.

7. Mamuye Y. Antibiotic Resistance Patterns of Common Gram-negative Uropathogens in St. Paul’s Hospital Millennium Medical College. Ethiop J Health Sci. 2016;26:93–100.

8. Nigussie D, Amsalu A. Prevalence of uropathogen and their antibiotic resistance pattern among diabetic patients. Turk Urol Derg. 2017;43:85–92.

9. Yeshitela B, Gebre-Selassie S, Feleke Y. Asymptomatic bacteriuria and symptomatic urinary tract infections (UTI) in patients with diabetes mellitus in Tikur Anbessa Specialized University Hospital, Addis Ababa, Ethiopia. Ethiop Med J. Ethiopia; 2012;50:239–49.

10. Merga Duffa Y, Terfa Kitila K, Mamuye Gebretsadik D, Bitew A. Prevalence and Antimicrobial Susceptibility of Bacterial Uropathogens Isolated from Pediatric Patients at Yekatit 12 Hospital Medical College, Addis Ababa, Ethiopia. Int J Microbiol. Hindawi; 2018;2018.

11. Blondeau JM, Vaughan D. A review of antimicrobial resistance in East Africa. Can J Microbiol. 2000;46:867–77.

12. Demilie T, Beyene G, Melaku S, Tsegaye W. Urinary bacterial profile and antibiotic susceptibility pattern among pregnant women in north west ethiopia. Ethiop J Health Sci. 2012;22:121–8.

13. Derese B, Kedir H, Teklemariam Z, Weldegebreal F, Balakrishnan S. Bacterial profile of urinary tract infection and antimicrobial susceptibility pattern among pregnant women attending at Antenatal Clinic in Dil Chora Referral Hospital, Dire Dawa, Eastern Ethiopia. Ther Clin Risk Manag. 2016;12:251–60.

14. Woldemariam HK, Geleta DA, Tulu KD, Aber NA, Legese MH, Fenta GM, et al. Common uropathogens and their antibiotic susceptibility pattern among diabetic patients. BMC Infect Dis. BMC Infectious Diseases; 2019;19:1–10.

15. Wondimeneh Y, Muluye D, Alemu A, Atinafu A, Yitayew G, Gebrecherkos T, et al. Urinary tract infection among obstetric fistula patients at Gondar University Hospital, Northwest Ethiopia. BMC Womens Health. 2014;14:2–7.

16. Tuem KB, Desta R, Bitew H, Ibrahim S, Hishe HZ. Antimicrobial resistance patterns of uropathogens isolated between 2012 and 2017 from a tertiary hospital in Northern Ethiopia. J Glob Antimicrob Resist. Taibah University; 2019;18:109–14.

17. Gessese YA, Damessa DL, Amare MM, Bahta YH, Shifera AD, Tasew FS, et al. Urinary pathogenic bacterial profile, antibiogram of isolates and associated risk factors among pregnant women in Ambo town, Central Ethiopia: A cross-sectional study. Antimicrob Resist Infect Control. Antimicrobial Resistance & Infection Control; 2017;6:1–10.

18. Bitew A, Molalign T, Chanie M. Species distribution and antibiotic susceptibility profile of bacterial uropathogens among patients complaining urinary tract infections. BMC Infect Dis. BMC Infectious Diseases; 2017;17:1–8.

19. Kibret M, Abera B. Prevalence and antibiogram of bacterial isolates from urinary tract infections at Dessie Health Research Laboratory, Ethiopia. Asian Pac J Trop Biomed. 2014;4:164–8.

20. Awoke N, Kassa T, Teshager L. Magnitude of Biofilm Formation and Antimicrobial Resistance Pattern of Bacteria Isolated from Urinary Catheterized Inpatients of Jimma University Medical Center, Southwest Ethiopia. Int J Microbiol. 2019;2019.

21. Gutema T, Weldegebreal F, Marami D, Teklemariam Z. Prevalence, antimicrobial susceptibility pattern, and associated factors of urinary tract infections among adult diabetic patients at Metu Karl Heinz Referral Hospital, Southwest Ethiopia. Int J Microbiol. 2018;2018.

22. Abejew AA, Denboba AA, Mekonnen AG. Prevalence and antibiotic resistance pattern of urinary tract bacterial infections in Dessie area, North-East Ethiopia. BMC Res Notes. 2014;7:1–7.

23. Marami D, Balakrishnan S, Seyoum B. Prevalence, Antimicrobial Susceptibility Pattern of Bacterial Isolates, and Associated Factors of Urinary Tract Infections among HIV-Positive Patients at Hiwot Fana Specialized University Hospital, Eastern Ethiopia. Can J Infect Dis Med Microbiol. 2019;2019.

24. Mitiku E, Amsalu A, Tadesse BT. Pediatric Urinary Tract Infection as a Cause of Outpatient Clinic Visits in Southern Ethiopia: A Cross Sectional Study. Ethiop J Health Sci. 2018;28:187–96.

25. Gashe F, Mulisa E, Mekonnen M, Zeleke G. Antimicrobial Resistance Profile of Different Clinical Isolates against Third-Generation Cephalosporins. J Pharm. 2018;2018:1–7.

26. Abebe W, Earsido A, Taye S, Assefa M, Eyasu A, Godebo G. Prevalence and antibiotic susceptibility patterns of Shigella and Salmonella among children aged below five years with Diarrhoea attending Nigist Eleni Mohammed memorial hospital, South Ethiopia. BMC Pediatr. BMC Pediatrics; 2018;18:10–5.

27. Adugna A, Kibret M, Abera B, Nibret E, Adal M. Antibiogram of e. Coli serotypes isolated from children aged under five with acute diarrhea in bahir dar town. Afr Health Sci. 2015;15:656–64.

28. Alemu A, Geta M, Taye S, Eshetie S, Engda T. Prevalence, associated risk factors and antimicrobial susceptibility patterns of Shigella infections among diarrheic pediatric population attending at Gondar town healthcare institutions, Northwest Ethiopia. Trop Dis Travel Med Vaccines. Tropical Diseases, Travel Medicine and Vaccines; 2019;5:1–8.

29. Eguale T, Gebreyes WA, Asrat D, Alemayehu H, Gunn JS, Engidawork E. Non-typhoidal Salmonella serotypes, antimicrobial resistance and co-infection with parasites among patients with diarrhea and other gastrointestinal complaints in Addis Ababa, Ethiopia. BMC Infect Dis. BMC Infectious Diseases; 2015;15:1–9.

30. Gebreegziabher G, Asrat D, W/Amanuel Y, Hagos T. Isolation and Antimicrobial Susceptibility Profile of Shigella and Salmonella Species from Children with Acute Diarrhoea in Mekelle Hospital and Semen Health Center, Ethiopia. Ethiop J Health Sci. 2018;28:197–206.

31. Gebrekidan A, Dejene TA, Kahsay G, Wasihun AG. Prevalence and antimicrobial susceptibility patterns of Shigella among acute diarrheal outpatients in Mekelle hospital, Northern Ethiopia. BMC Res Notes. BioMed Central; 2015;8:1–7.

32. Gebresilasie YM, Tullu KD, Yeshanew AG. Resistance pattern and maternal knowledge, attitude and practices of suspected Diarrheagenic Escherichia coli among children under 5 years of age in Addis Ababa, Ethiopia: Cross sectional study. Antimicrob Resist Infect Control. Antimicrobial Resistance & Infection Control; 2018;7:1–9.

33. Lamboro T, Ketema T, Bacha K. Prevalence and Antimicrobial Resistance in Salmonella and Shigella Species Isolated from Outpatients, Jimma University Specialized Hospital, Southwest Ethiopia. Can J Infect Dis Med Microbiol. Hindawi Publishing Corporation; 2016;2016.

34. Mulatu G, , Beyene, G, Zeynudin A. Prevalence of Salmonella and Cmpylobacter Species and Their Susceptibility Patters Among Under Five Children With Diarrhea. Ethiop J Heal Sci. 2014;24.

35. Terfassa A, Jida M. Prevalence and antibiotics susceptibility pattern of salmonella and shigella species among diarrheal patients attending nekemte referral hospital, oromia, Ethiopia. Int J Microbiol. 2018;2018.

36. Debas G, Kibret M, Biadglegne F, Abera B. PRevalence and antimicrobial susceptibility patterns of shigella species at felege hiwot referral hospital, northwest ethiopia. Ethiop Med J. 2011;49:249–56.

37. Ewnetu D, Mihret A. Prevalence and antimicrobial resistance of Campylobacter isolates from humans and chickens in Bahir Dar, Ethiopia. Foodborne Pathog Dis. Mary Ann Liebert, Inc. 140 Huguenot Street, 3rd Floor New Rochelle, NY 10801 USA; 2010;7:667–70.

38. Lengerh A, Moges F, Unakal C, Anagaw B. Prevalence, associated risk factors and antimicrobial susceptibility pattern of Campylobacter species among under five diarrheic children at Gondar University Hospital, Northwest Ethiopia. BMC Pediatr. BMC Pediatrics; 2013;13:82.

39. Tafa B, Sewunet T, Tassew H, Asrat D. Isolation and Antimicrobial Susceptibility Patterns of Campylobacter Species among Diarrheic Children at Jimma, Ethiopia . Int J Bacteriol. Hindawi Publishing Corporation; 2014;2014:1–7.

40. Teshome B, Teklemariam Z, Admassu Ayana D, Marami D, Asaminew N. Salmonella and Shigella among patients with diarrhea at public health facilities in Adama, Ethiopia: Prevalence, antimicrobial susceptibility pattern, and associated factors . SAGE Open Med. 2019;7:205031211984604.

41. Zelelie TZ, Gebreyes DS, Tilahun AT, Craddock HA, Gishen NZ. Enteropathogens in Under-Five Children with Diarrhea in Health Facilities of Debre Berhan Town, North Shoa, Ethiopia. Ethiop J Health Sci. 2019;29:203–14.

42. Eguale T, Asrat D, Alemayehu H, Nana I, Gebreyes WA, Gunn JS, et al. Phenotypic and genotypic characterization of temporally related nontyphoidal Salmonella strains isolated from humans and food animals in central Ethiopia. Zoonoses Public Health. 2018;65:766–76.

43. Abera B, Bezabih B, Dessie A. Antimicrobial suceptibility of V. cholerae in north west, Ethiopia. Ethiop Med J. 2010;48:23–8.

44. Mohammed A, Seid ME, Gebrecherkos T, Tiruneh M, Moges F. Bacterial Isolates and Their Antimicrobial Susceptibility Patterns of Wound Infections among Inpatients and Outpatients Attending the University of Gondar Referral Hospital, Northwest Ethiopia. Int J Microbiol. 2017;2017.

45. Mulualem Y, Kasa T, Mekonnen Z, Suleman S. Occurrence of extended spectrum beta (b)-lactamases in multi-drug resistant Escherichia coli isolated from a clinical setting in Jimma University Specialized Hospital, Jimma, southwest Ethiopia. East Afr J Public Health. 2012;9:58–61.

46. Mama M, Teshome T, Detamo J. Antibacterial Activity of Honey against Methicillin-Resistant Staphylococcus aureus: A Laboratory-Based Experimental Study. Int J Microbiol. 2019;2019.

47. Sewunet T, Demissie Y, Mihret A, Abebe T. Bacterial profile and antimicrobial susceptibility pattern of isolates among burn patients at Yekatit 12 Hospital Burn Center, Addis Ababa, Ethiopia. Ethiop J Health Sci. 2013;23:209–16.

48. Azene MK, Beyene BA. Bacteriology and antibiogram of pathogens from wound infections at Dessie Laboratory, North East Ethiopia. Tanzan J Health Res. 2011;13.

49. Godebo G, Kibru G, Tassew H. Multidrug-resistant bacterial isolates in infected wounds at Jimma University Specialized Hospital, Ethiopia. Ann Clin Microbiol Antimicrob. Annals of Clinical Microbiology and Antimicrobials; 2013;12:1.

50. Mama M, Abdissa A, Sewunet T. Antimicrobial susceptibility pattern of bacterial isolates from wound infection and their sensitivity to alternative topical agents at Jimma University Specialized Hospital, South-West Ethiopia. Ann Clin Microbiol Antimicrob. 2014;13:1–10.

51. Hailegiyorgis TT, Sarhie WD, Workie HM. Isolation and antimicrobial drug susceptibility pattern of bacterial pathogens from pediatric patients with otitis media in selected health institutions, Addis Ababa, Ethiopia: A prospective cross-sectional study. BMC Ear, Nose Throat Disord. BMC Ear, Nose and Throat Disorders; 2018;18:1–7.

52. Deyno S, Fekadu S, Astatkie A. Resistance of Staphylococcus aureus to antimicrobial agents in Ethiopia: a meta-analysis. Antimicrob Resist Infect Control. Springer; 2017;6:85.

53. Hailu D, Mekonnen D, Derbie A, Mulu W, Abera B. Pathogenic bacteria profile and antimicrobial susceptibility patterns of ear infection at Bahir Dar Regional Health Research Laboratory Center, Ethiopia. Springerplus. Springer International Publishing; 2016;5.

54. Muluye D, Wondimeneh Y, Moges F, Nega T, Ferede G. Types and drug susceptibility patterns of bacterial isolates from eye discharge samples at Gondar University Hospital, Northwest Ethiopia. BMC Res Notes. 2014;7:1–5.

55. Gorems K, Beyene G, Berhane M, Mekonnen Z. Antimicrobial susceptibility patterns of bacteria isolated from patients with ear discharge in Jimma Town, Southwest, Ethiopia. BMC Ear, Nose Throat Disord. BMC Ear, Nose and Throat Disorders; 2018;18:1–9.

56. Wasihun AG, Zemene Y. Bacterial profile and antimicrobial susceptibility patterns of otitis media in Ayder Teaching and Referral Hospital, Mekelle University, Northern Ethiopia. Springerplus. Springer International Publishing; 2015;4:1–9.

57. Argaw-Denboba A, Abejew AA, Mekonnen AG. Antibiotic-resistant bacteria are major threats of otitis media in wollo area, northeastern Ethiopia: A ten-year retrospective analysis. Int J Microbiol. Hindawi Publishing Corporation; 2016;2016.

58. Abera B, Biadeglegne F. Antimicrobial resistance patterns of staphylococcus aureus and Proteus spp. Isolated from otitis media at Bahir Dar regional laboratory, North West Ethiopia. Ethiop Med J. 2009;47:271–6.

59. Shiferaw B, Gelaw B, Assefa A, Assefa Y, Addis Z. Bacterial isolates and their antimicrobial susceptibility pattern among patients with external ocular infections at Borumeda hospital, Northeast Ethiopia. BMC Ophthalmol. BMC Ophthalmology; 2015;15:1–8.

60. Assefa Y, Moges F, Endris M, Zereay B, Amare B, Bekele D, et al. Bacteriological profile and drug susceptibility patterns in dacryocystitis patients attending Gondar University Teaching Hospital, Northwest Ethiopia Inflammatory eye diseases. BMC Ophthalmol. 2015;15:1–8.

61. Muluye D, Wondimeneh Y, Ferede G, Moges F, Nega T. Bacterial isolates and drug susceptibility patterns of ear discharge from patients with ear infection at Gondar University Hospital, Northwest Ethiopia. BMC Ear, Nose Throat Disord. BMC Ear, Nose and Throat Disorders; 2013;13:1.

62. Belyhun Y, Moges F, Endris M, Asmare B, Amare B, Bekele D, et al. Ocular bacterial infections and antibiotic resistance patterns in patients attending Gondar Teaching Hospital, Northwest Ethiopia. BMC Res Notes. BioMed Central; 2018;11:1–7.

63. Teweldemedhin M, Saravanan M, Gebreyesus A, Gebreegziabiher D. Ocular bacterial infections at Quiha Ophthalmic Hospital, Northern Ethiopia: An evaluation according to the risk factors and the antimicrobial susceptibility of bacterial isolates. BMC Infect Dis. BMC Infectious Diseases; 2017;17:1–11.

64. Tamrat L, Gelaw Y, Beyene G, Gize A. Microbial Contamination and Antimicrobial Resistance in Use of Ophthalmic Solutions at the Department of Ophthalmology, Jimma University Specialized Hospital, Southwest Ethiopia. Can J Infect Dis Med Microbiol. 2019;2019:6–8.

65. Getahun E, Gelaw B, Assefa A, Assefa Y, Amsalu A. Bacterial pathogens associated with external ocular infections alongside eminent proportion of multidrug resistant isolates at the University of Gondar Hospital, northwest Ethiopia. BMC Ophthalmol. BMC Ophthalmology; 2017;17:1–10.

66. Dessie W, Mulugeta G, Fentaw S, Mihret A, Hassen M, Abebe E. Pattern of bacterial pathogens and their susceptibility isolated from surgical site infections at selected referral hospitals, Addis Ababa, Ethiopia. Int J Microbiol. 2016;2016.

67. Mulu W, Kibru G, Beyene G, Damtie M. Postoperative Nosocomial Infections and Antimicrobial Resistance Pattern of Bacteria Isolates among Patients Admitted at Felege Hiwot Referral Hospital, Bahirdar, Ethiopia. Ethiop J Health Sci. 2012;22:7–18.

68. Gashaw M, Berhane M, Bekele S, Kibru G, Teshager L, Yilma Y, et al. Emergence of high drug resistant bacterial isolates from patients with health care associated infections at Jimma University medical center: A cross sectional study. Antimicrob Resist Infect Control. Antimicrobial Resistance & Infection Control; 2018;7:1–8.

69. Kifilie AB, Dagnew M, Tegenie B, Yeshitela B, Howe R, Abate E. Bacterial Profile, Antibacterial Resistance Pattern, and Associated Factors from Women Attending Postnatal Health Service at University of Gondar Teaching Hospital, Northwest Ethiopia. Int J Microbiol. Hindawi; 2018;2018:1–10.

70. Mengesha RE, Kasa BGS, Saravanan M, Berhe DF, Wasihun AG. Aerobic bacteria in post surgical wound infections and pattern of their antimicrobial susceptibility in Ayder Teaching and Referral Hospital, Mekelle, Ethiopia. BMC Res Notes. 2014;7:4–9.

71. Kahsay A, Mihret A, Abebe T, Andualem T. Isolation and antimicrobial susceptibility pattern of Staphylococcus aureus in patients with surgical site infection at Debre Markos Referral Hospital, Amhara Region, Ethiopia. Arch Public Heal. 2014;72:1–7.

72. Seboxa T, Amogne W, Abebe W, Tsegaye T, Azazh A, Hailu W, et al. High mortality from blood stream infection in Addis Ababa, Ethiopia, is due to antimicrobial resistance. PLoS One. 2015;10:1–14.

73. Alebachew G, Teka B, Endris M, Shiferaw Y, Tessema B. Etiologic Agents of Bacterial Sepsis and Their Antibiotic Susceptibility Patterns among Patients Living with Human Immunodeficiency Virus at Gondar University Teaching Hospital, Northwest Ethiopia. Biomed Res Int. Hindawi Publishing Corporation; 2016;2016.

74. Negussie A, Mulugeta G, Bedru A, Ali I, Shimeles D, Lema T, et al. Bacteriological Profile and Antimicrobial Susceptibility Pattern of Blood Culture Isolates among Septicemia Suspected Children in Selected Hospitals Addis Ababa, Ethiopia. Int J Biol Med Res. 2015;6:4709–17.

75. Sorsa A, Früh J, Stötter L, Abdissa S. Blood culture result profile and antimicrobial resistance pattern: A report from neonatal intensive care unit (NICU), Asella teaching and referral hospital, Asella, south East Ethiopia. Antimicrob Resist Infect Control. Antimicrobial Resistance & Infection Control; 2019;8:6–11.

76. Geyesus T, Moges F, Eshetie S, Yeshitela B, Abate E. Bacterial etiologic agents causing neonatal sepsis and associated risk factors in Gondar, Northwest Ethiopia. BMC Pediatr. BMC Pediatrics; 2017;17:1–10.

77. Adhanom G, Gebreegziabiher D, Weldu Y, Gebreyesus Wasihun A, Araya T, Legese H, et al. Species, Risk Factors, and Antimicrobial Susceptibility Profiles of Bacterial Isolates from HIV-Infected Patients Suspected to Have Pneumonia in Mekelle Zone, Tigray, Northern Ethiopia. Biomed Res Int. 2019;2019:1–10.

78. Negash AA, Asrat D, Abebe W, Hailemariam T, Hailu T, Aseffa A, et al. Bacteremic community-acquired pneumonia in Ethiopian children: Etiology, antibiotic resistance, risk factors, and clinical outcome. Open Forum Infect Dis. 2019;6:1–8.

79. Temesgen D, Bereded F, Derbie A, Biadglegne F. Bacteriology of community acquired pneumonia in adult patients at Felege Hiwot Referral Hospital, Northwest Ethiopia: A cross-sectional study. Antimicrob Resist Infect Control. Antimicrobial Resistance & Infection Control; 2019;8:1–8.

80. Ali S, Sewunet T, Sahlemariam Z, Kibru G. Neisseria gonorrhoeae among suspects of sexually transmitted infection in Gambella hospital, Ethiopia: Risk factors and drug resistance. BMC Res Notes. BioMed Central; 2016;9:1–8.

81. Hailemariam M, Abebe T, Mihret A, Lambiyo T. Prevalence of Neisseria gonorrhea and their antimicrobial susceptibility patterns among symptomatic women attending gynecology outpatient department in Hawassa referral hospital, Hawassa, Ethiopia. Ethiop J Health Sci. 2013;23:10–8.

82. Yeshanew AG, Geremew RA. Neisseria Gonorrhoae and their antimicrobial susceptibility patterns among symptomatic patients from Gondar town, north West Ethiopia. Antimicrob Resist Infect Control. Antimicrobial Resistance & Infection Control; 2018;7:1–7.

83. Mulu W, Yimer M, Zenebe Y, Abera B. Common causes of vaginal infections and antibiotic susceptibility of aerobic bacterial isolates in women of reproductive age attending at Felegehiwot referral Hospital, Ethiopia: A cross sectional study. BMC Womens Health. ???; 2015;15:1–9.

84. Bitew A, Abebaw Y, Bekele D, Mihret A. Prevalence of bacterial vaginosis and associated risk factors among women complaining of genital tract infection. Int J Microbiol. Hindawi; 2017;2017.

85. Desta K, Woldeamanuel Y, Azazh A, Mohammod H, Desalegn D, Shimelis D, et al. High gastrointestinal colonization rate with extended-spectrum β-lactamase-producing Enterobacteriaceae in hospitalized patients: Emergence of carbapenemase-producing K. Pneumoniae in Ethiopia. PLoS One. 2016;11:1–14.

86. Kebede A, Aragie S, Shimelis T. The common enteric bacterial pathogens and their antimicrobial susceptibility pattern among HIV-infected individuals attending the antiretroviral therapy clinic of Hawassa university hospital, southern Ethiopia. Antimicrob Resist Infect Control. Antimicrobial Resistance & Infection Control; 2017;6:1–7.

87. Abera B, Kibret M. Azithromycin, fluoroquinolone and chloramphenicol resistance of non-chlamydia conjunctival bacteria in rural community of Ethiopia. Indian J Ophthalmol. 2014;62:236–9.

88. Fentie A, Wondimeneh Y, Balcha A, Amsalu A, Adankie BT. Bacterial profile, antibiotic resistance pattern and associated factors among cancer patients at university of Gondar Hospital, northwest Ethiopia. Infect Drug Resist. 2018;11:2169–78.

89. Arega B, Wolde-Amanuel Y, Adane K, Belay E, Abubeker A, Asrat D. Rare bacterial isolates causing bloodstream infections in Ethiopian patients with cancer. Infect Agent Cancer. Infectious Agents and Cancer; 2017;12:4–9.

90. Arega B, Woldeamanuel Y, Adane K, Sherif AA, Asrat D. Microbial spectrum and drug-resistance profile of isolates causing bloodstream infections in febrile cancer patients at a referral hospital in Addis Ababa, Ethiopia. Infect Drug Resist. 2018;11:1511–9.

91. Deksissa T, Gebremedhin EZ. A cross-sectional study of enteric fever among febrile patients at Ambo hospital: Prevalence, risk factors, comparison of Widal test and stool culture and antimicrobials susceptibility pattern of isolates. BMC Infect Dis. BMC Infectious Diseases; 2019;19:1–12.

92. Marks F, von Kalckreuth V, Aaby P, Adu-Sarkodie Y, El Tayeb MA, Ali M, et al. Incidence of invasive salmonella disease in sub-Saharan Africa: a multicentre population-based surveillance study. Lancet Glob Heal. 2017;5:e310–23.

93. Zenebe T, Kannan S, Yilma D, Beyene G. Original Article Invasive Bacterial Pathogens and Their Antibiotic Susceptibility Patterns in Jimma Specialized Hospital ,. 2011;1–8.

94. Wasihun AG, Wlekidan LN, Gebremariam SA, Dejene TA, Welderufael AL, Haile TD, et al. Bacteriological profile and antimicrobial susceptibility patterns of blood culture isolates among febrile patients in mekelle hospital, Northern Ethiopia. Springerplus. Springer International Publishing; 2015;4.

95. wasihun AG, Wlekidan LN, Gebremariam SA, Welderufael AL, Muthupandian S, Haile TD, et al. Diagnosis and Treatment of Typhoid Fever and Associated Prevailing Drug Resistance in Northern Ethiopia. Int J Infect Dis. International Society for Infectious Diseases; 2015;35:e96–102.

96. Mitiku H, Weldegebreal F, Marami D, Teklemariam Z. Nontyphoidal salmonella bacteremia in antiretroviral therapy-naïve HIV-infected individuals at three public hospitals in eastern Ethiopia: Prevalence, antimicrobial susceptibility patterns, and associated factors. HIV/AIDS - Res Palliat Care. 2019;11:23–9.

97. Lemma MT, Zenebe Y, Tulu B, Mekonnen D, Mekonnen Z. Methicillin resistant staphylococcus aureus among HIV infected pediatric patients in northwest Ethiopia: Carriage rates and antibiotic co-resistance profiles. PLoS One. 2015;10:1–10.

98. Tolera M, Abate D, Dheresa M, Marami D. Bacterial Nosocomial Infections and Antimicrobial Susceptibility Pattern among Patients Admitted at Hiwot Fana Specialized University Hospital, Eastern Ethiopia. Adv Med. 2018;2018:1–7.

99. Feleke T, Eshetie S, Dagnew M, Endris M, Abebe W, Tiruneh M, et al. Multidrug-resistant bacterial isolates from patients suspected of nosocomial infections at the University of Gondar Comprehensive Specialized Hospital, Northwest Ethiopia. BMC Res Notes. BioMed Central; 2018;11:1–7.

100. Anagaw B, Gezachew M, Biadgelgene F, Anagaw B, Geleshe T, Taddese B, et al. Antimicrobial susceptibility patterns of Streptococcus pneumoniae over 6 years at Gondar University Hospital, Northwest Ethiopia. Asian Pac J Trop Biomed. 2013;3:536–41.

101. Assefa A, Gelaw B, Shiferaw Y, Tigabu Z. Nasopharyngeal carriage and antimicrobial susceptibility pattern of streptococcus pneumoniae among pediatric outpatients at gondar university hospital, north west ethiopia. Pediatr Neonatol. Elsevier Taiwan LLC; 2013;54:315–21.

102. Assefa S, Desta K, Lema T. Group B streptococci vaginal colonization and drug susceptibility pattern among pregnant women attending in selected public antenatal care centers in Addis Ababa, Ethiopia. BMC Pregnancy Childbirth. BMC Pregnancy and Childbirth; 2018;18:1–9.

103. Gizachew M, Tiruneh M, Moges F, Adefris M, Tigabu Z, Tessema B. Streptococcus agalactiae from Ethiopian pregnant women; Prevalence, associated factors and antimicrobial resistance: Alarming for prophylaxis. Ann Clin Microbiol Antimicrob. BioMed Central; 2019;18:1–9.

104. Mengist HM, Zewdie O, Belew A, Dabsu R. Prevalence and drug susceptibility pattern of group B Streptococci (GBS) among pregnant women attending antenatal care (ANC) in Nekemte Referral Hospital (NRH), Nekemte, Ethiopia. BMC Res Notes. BioMed Central; 2017;10:388.

105. Toru M, Beyene G, Kassa T, Gizachew Z, Howe R, Yeshitila B. Prevalence and phenotypic characterization of Enterococcus species isolated from clinical samples of pediatric patients in Jimma University Specialized Hospital, south west Ethiopia. BMC Res Notes. BioMed Central; 2018;11.

106. Abdissa A, Asrat D, Shitu B, Zeidan M, Kronvall G, Achiko D, et al. Erratum: Throat carriage rate and antimicrobial susceptibility pattern of group a streptococci (Gas) in healthy ethiopian school children (Ethiopia Journal of Medicine (2011) 49: 2 (125)). Ethiop Med J. 2011;49:283.

107. Tadesse S, Kahsay T, Adhanom G, Kahsu G, Legese H, Gwahid A, et al. Prevalence, antimicrobial susceptibility profile and predictors of asymptomatic bacteriuria among pregnant women in Adigrat General Hospital, Northern Ethiopia. BMC Res Notes. BioMed Central; 2018;11:1–6.

108. Weldegebreal F, Admassu D, Meaza D, Asfaw M. Non-critical healthcare tools as a potential source of healthcare-acquired bacterial infections in eastern Ethiopia: A hospital-based cross-sectional study. SAGE Open Med. 2019;7:205031211882262.

109. Genet C, Kibru G, Tsegaye W. Indoor Air Bacterial Load and Antibiotic Susceptibility Pattern of Isolates in Operating Rooms and Surgical Wards at Jimma University Specialized Hospital, Southwest Ethiopia. Ethiop J Health Sci. 2011;21:9–17.

110. Dilnessa T, Bitew A. Prevalence and antimicrobial susceptibility pattern of methicillin resistant Staphylococcus aureus isolated from clinical samples at Yekatit 12 Hospital Medical College, Addis Ababa, Ethiopia. BMC Infect Dis. BMC Infectious Diseases; 2016;16:1–9.

111. Abera B, Kibret M, Mulu W. Extended-spectrum beta (β)-lactamases and antibiogram in enterobacteriaceae from clinical and drinking water sources from bahir dar city, Ethiopia. PLoS One. 2016;11:1–10.

112. Alemayehu T, Mekasha A, Abebe T. Nasal carriage rate and antibiotic susceptibility pattern of Neisseria meningitidis in healthy Ethiopian children and adolescents: A cross-sectional study. PLoS One. 2017;12:1–11.

113. Agzie M, Niguse S, Tsegay E, Kahsay G, Mahmud MA. Bacterial contaminants of stored blood and blood components ready for transfusion at blood banks in Mekelle, Northern Ethiopia. BMC Res Notes. BioMed Central; 2019;12:1–6.

114. Dagnew M, Tiruneh M, Moges F, Tekeste Z. Survey of nasal carriage of Staphylococcus aureus and intestinal parasites among food handlers working at Gondar University, Northwest Ethiopia. BMC Public Health. BMC Public Health; 2012;12:1.

115. Kahsay AG, Hagos DG, Abay GK, Mezgebo TA. Prevalence and antimicrobial susceptibility patterns of methicillin-resistant Staphylococcus aureus among janitors of Mekelle University, North Ethiopia. BMC Res Notes. BioMed Central; 2018;11:1–6.

116. Getachew H, Derbie A, Mekonnen D. Surfaces and Air Bacteriology of Selected Wards at a Referral Hospital, Northwest Ethiopia: A Cross-Sectional Study. Int J Microbiol. 2018;2018.

117. Birri DJ, Brede DA, Tessema GT, Nes IF. Bacteriocin Production, Antibiotic Susceptibility and Prevalence of Haemolytic and Gelatinase Activity in Faecal Lactic Acid Bacteria Isolated from Healthy Ethiopian Infants. Microb Ecol. 2013;65:504–16.

118. Engda T, Moges F, Gelaw A, Eshete S, Mekonnen F. Prevalence and antimicrobial susceptibility patterns of extended spectrum beta-lactamase producing Entrobacteriaceae in the University of Gondar Referral Hospital environments, northwest Ethiopia. BMC Res Notes. BioMed Central; 2018;11:1–7.

119. Garedew-Kifelew L, Wondafrash N, Feleke A. Identification of drug-resistant Salmonella from food handlers at the University of Gondar, Ethiopia. BMC Res Notes. 2014;7:1–6.

120. Gizachew M, Tiruneh M, Moges F, Adefris M, Tigabu Z, Tessema B. Newborn colonization and antibiotic susceptibility patterns of Streptococcus agalactiae at the University of Gondar Referral Hospital, Northwest Ethiopia 11 Medical and Health Sciences 1117 Public Health and Health Services. BMC Pediatr. BMC Pediatrics; 2018;18:1–11.

121. Wada FW, Tufa EG, Berheto TM, Solomon FB. Nasopharyngeal carriage of Streptococcus pneumoniae and antimicrobial susceptibility pattern among school children in South Ethiopia: Post-vaccination era. BMC Res Notes. BioMed Central; 2019;12:1–6.

122. Zeynudin A, Pritsch M, Schubert S, Messerer M, Liegl G, Hoelscher M, et al. Prevalence and antibiotic susceptibility pattern of CTX-M type extended-spectrum β-lactamases among clinical isolates of gram-negative bacilli in Jimma, Ethiopia. BMC Infect Dis. BMC Infectious Diseases; 2018;18:1–10.

123. Solomon FB, Wadilo F, Tufa EG, Mitiku M. Extended spectrum and metalo beta-lactamase producing airborne Pseudomonas aeruginosa and Acinetobacter baumanii in restricted settings of a referral hospital: A neglected condition. Antimicrob Resist Infect Control. Antimicrobial Resistance & Infection Control; 2017;6:1–7.

124. Wondimu H, Addis Z, Moges F, Shiferaw Y. Bacteriological Safety of Blood Collected for Transfusion at University of Gondar Hospital Blood Bank, Northwest Ethiopia. ISRN Hematol. 2013;2013:1–7.

125. Reta A, Wubie M, Mekuria G. Nasal colonization and antimicrobial susceptibility pattern of Staphylococcus aureus among pre-school children in Ethiopia. BMC Res Notes. BioMed Central; 2017;10:1–7.

126. Legese H, Kahsay AG, Kahsay A, Araya T, Adhanom G, Muthupandian S, et al. Nasal carriage, risk factors and antimicrobial susceptibility pattern of methicillin resistant Staphylococcus aureus among healthcare workers in Adigrat and Wukro hospitals, Tigray, Northern Ethiopia. BMC Res Notes. BioMed Central; 2018;11:1–6.

127. Shibabaw A, Abebe T, Mihret A. Antimicrobial susceptibility pattern of nasal Staphylococcus aureus among Dessie Referral Hospital health care workers, Dessie, Northeast Ethiopia. Int J Infect Dis. International Society for Infectious Diseases; 2014;25:22–5.

128. Solomon FB, Wadilo FW, Arota AA, Abraham YL. Antibiotic resistant airborne bacteria and their multidrug resistance pattern at University teaching referral Hospital in South Ethiopia. Ann Clin Microbiol Antimicrob. BioMed Central; 2017;16:1–7.

129. Mulu W, Abera B, Yimer M, Hailu T, Ayele H, Abate D. Bacterial agents and antibiotic resistance profiles of infections from different sites that occurred among patients at Debre Markos Referral Hospital, Ethiopia: A cross-sectional study. BMC Res Notes. BioMed Central; 2017;10:1–9.

130. Tigabu A, Tiruneh M, Mekonnen F. Nasal Carriage Rate, Antimicrobial Susceptibility Pattern, and Associated Factors of Staphylococcus aureus with Special Emphasis on MRSA among Urban and Rural Elementary School Children in Gondar, Northwest Ethiopia: A Comparative Cross-Sectional Study . Adv Prev Med. 2018;2018:1–11.

131. Kejela T, Bacha K. Prevalence and antibiotic susceptibility pattern of methicillin-resistant Staphylococcus aureus (MRSA) among primary school children and prisoners in Jimma Town, Southwest Ethiopia. Ann Clin Microbiol Antimicrob. Annals of Clinical Microbiology and Antimicrobials; 2013;12:1.
